# *ent*-Beyerane Diterpenes as a Key Platform for the Development of ArnT-Mediated Colistin Resistance Inhibitors

**DOI:** 10.1021/acs.joc.0c01459

**Published:** 2020-07-27

**Authors:** Deborah Quaglio, Maria Luisa Mangoni, Roberta Stefanelli, Silvia Corradi, Bruno Casciaro, Valeria Vergine, Federica Lucantoni, Luca Cavinato, Silvia Cammarone, Maria Rosa Loffredo, Floriana Cappiello, Andrea Calcaterra, Silvia Erazo, Francesca Ghirga, Mattia Mori, Francesco Imperi, Fiorentina Ascenzioni, Bruno Botta

**Affiliations:** † Department of Chemistry and Technology of Drugs, “Department of Excellence 2018−2022”, Sapienza University of Rome, P.le Aldo Moro 5, 00185 Rome, Italy; ‡ Laboratory Affiliated to Pasteur Italia-Fondazione Cenci Bolognetti, Department of Biochemical Sciences, Sapienza University of Rome, P.le Aldo Moro 5, 00185 Rome, Italy; § Department of Biology and Biotechnology Charles Darwin, Sapienza University of Rome, Laboratory Affiliated to Pasteur Italia-Fondazione Cenci Bolognetti, Via dei Sardi 70, 00185 Rome, Italy; ∥ Department of Science, Roma Tre University, Viale Guglielmo Marconi 446, 00146 Rome, Italy; ⊥ Center for Life Nano Science@Sapienza, Istituto Italiano di Tecnologia, Viale Regina Elena, 291, 00161 Rome, Italy; # Department of Pharmacological and Toxicological Chemistry, Faculty of Chemical and Pharmaceutical Sciences, University of Chile, 1058 Santiago, Chile; ¶ Department of Biotechnology, Chemistry and Pharmacy, “Department of Excellence 2018−2022”, University of Siena, via Aldo Moro 2, 53100 Siena, Italy

## Abstract

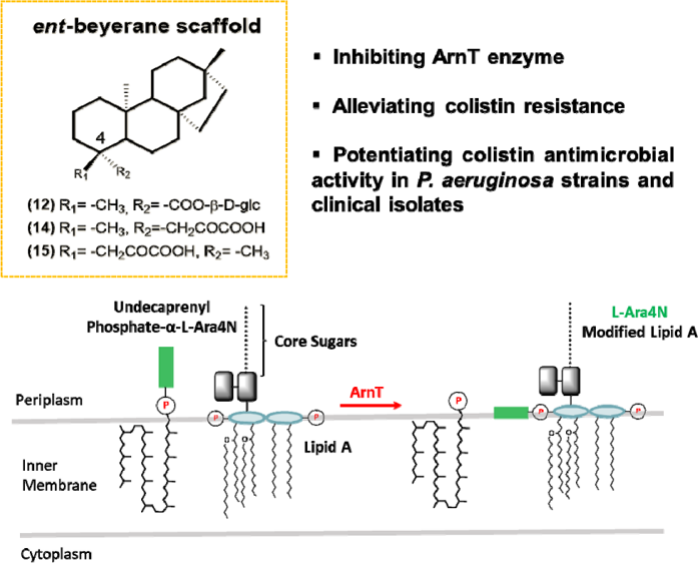

Colistin is a last-resort antibiotic for the treatment of multidrug resistant Gram-negative bacterial infections. Recently, a natural *ent*-beyerene diterpene was identified as a promising inhibitor of the enzyme responsible for colistin resistance mediated by lipid A aminoarabinosylation in Gram-negative bacteria, namely, ArnT (undecaprenyl phosphate-alpha-4-amino-4-deoxy-l-arabinose arabinosyl transferase). Here, semisynthetic analogues of hit were designed, synthetized, and tested against colistin-resistant *Pseudomonas aeruginosa* strains including clinical isolates to exploit the versatility of the diterpene scaffold. Microbiological assays coupled with molecular modeling indicated that for a more efficient colistin adjuvant activity, likely resulting from inhibition of the ArnT activity by the selected compounds and therefore from their interaction with the catalytic site of ArnT, an *ent*-beyerane scaffold is required along with an oxalate-like group at C-18/C-19 or a sugar residue at C-19 to resemble L-Ara4N. The *ent*-beyerane skeleton is identified for the first time as a privileged scaffold for further cost-effective development of valuable colistin resistance inhibitors.

## Introduction

Control of infections has long been a serious clinical concern and the discovery of antibiotics during the 1930s to 1960s opened the door to current antimicrobial drug discovery.^1^ Nevertheless, the excessive use of antibiotics in humans and in the livestock, the poor sanitation, and the release of nonmetabolized antibiotics in the surroundings have threatened most of the recorded advances.^2^ Together with the unavailability of newer drugs, these factors have contributed to the genetic selection pressure for the appearance and evolution of multidrug-resistant (MDR) bacteria with global spread in the last decades, which stand for a serious public health emergency and a current challenge with considerable economic impacts.^3,4^ As an example of MDR microorganisms, the Gram-negative bacterium *Pseudomonas aeruginosa* is one of the leading causes of nosocomial and chronic infections, especially in cystic fibrosis patients where it concurs to lung disease which accounts for more than 85% mortality.^5^*P. aeruginosa* has intrinsic resistance to a large number of antibiotics because of the low permeability of its outer membrane (OM), the presence of active efflux pumps, and the expression of antibiotic-modifying enzymes.^6^ Furthermore, at the infectious site, it often lives within biofilm communities that make bacteria recalcitrant to stressful environmental conditions, antibiotic treatments, and the host immune clearance.^7^ Currently, there are very few antipseudomonal agents in clinical development, while the lack of treatment options for MDR bacteria has contributed to reconsider colistin as a last-line antimicrobial therapy, despite its toxicity for kidneys and neural tissues.^8,9^ Colistin is a cationic multicomponent lipopeptide that targets lipopolysaccharides (LPS) in the OM of Gram-negative bacteria.^10,11^ It initially interacts with the anionic phosphate headgroups of the lipid A moiety of LPS, displacing divalent cations, that is, Ca^++^ and Mg^++^ that stabilize adjacent LPS molecules. This is then followed by the destabilization of the OM with subsequent disruption of the inner membrane, leading to cell death. Unfortunately, resistance to colistin has been documented in several case reports.^12,13^ This can have devastating effects if no other therapeutic strategies are uncovered to combat infections, including those associated with *P. aeruginosa* in cystic fibrosis lungs. One of the mechanisms of resistance consists in the covalent modification of LPS by the addition of 4-amino-4-deoxy-l-arabinose (L-Ara4N) or phosphoethanolamine groups to lipid A, which decreases the overall charge of LPS and, as a result, the binding affinity of the cationic lipopeptide.^14^ In *P. aeruginosa,* these changes are controlled by enzymes encoded by the *arn* operon which is regulated by several two-component systems. One of these enzymes is the glycosyltransferase ArnT (undecaprenyl phosphate-alpha-4-amino-4-deoxy-l-arabinose arabinosyl transferase), which catalyzes the transfer of L-Ara4N, provided by the lipid carrier undecaprenyl phosphate to lipid A phosphate groups.^15^ Potentiating the effect of existing antimicrobial compounds represents a promising approach to address the current antibiotics crisis and poor efficacy.^16^ In particular, inhibitors of resistance enzymes offer an alternative avenue to withstand this threat.^17^ The combination of such inhibitors with clinically relevant antibiotics may effectively extend the lifetime of these antibacterial drugs and minimize the impact of the appearance of resistance. Medicinal plants are an extraordinary rich storehouse of bioactive secondary metabolites with a large spectrum of enzyme inhibitory potential.^18−21^ They can work as ligands and bind to an enzyme blocking its activity with an irreversible or reversible process. Recently, a unique *in-house* library of natural products available in our group was screened *in silico* against the catalytic site of the ArnT enzyme to identify putative inhibitors of the Ara4N-dependent colistin resistance mechanism.^22^ This led to the selection of the *ent*-beyerene diterpene **1** (formerly known as BBN149), isolated from the leaves of *Fabiana densa* var. *ramulosa*, with a colistin adjuvant activity *versus* colistin-resistant *P. aeruginosa* strains, without any significant effect on colistin-susceptible strains.^22,23^ Here, we exploit the versatility of the diterpene scaffold by designing, synthesizing, and testing several analogues of **1**. Through the combination of computational modeling, organic synthesis, and biological evaluations in a concerted multidisciplinary strategy, we explore structure–activity relationships (SAR) around the initial diterpene hit **1** and validate its scaffold for the production of novel antibacterial agents for the treatment of colistin-resistant infections. Chemical analogues featuring a structurally related diterpene core were synthesized and screened *in vitro* against colistin-resistant *P. aeruginosa* strains including clinical isolates, while the putative binding mode against the ArnT enzyme was investigated by molecular modeling. Herein, the *ent*-beyerane skeleton is identified for the first time as a privileged scaffold for further development and optimization of valuable colistin resistance inhibitors.

## Results and Discussion

**Chart 1:**
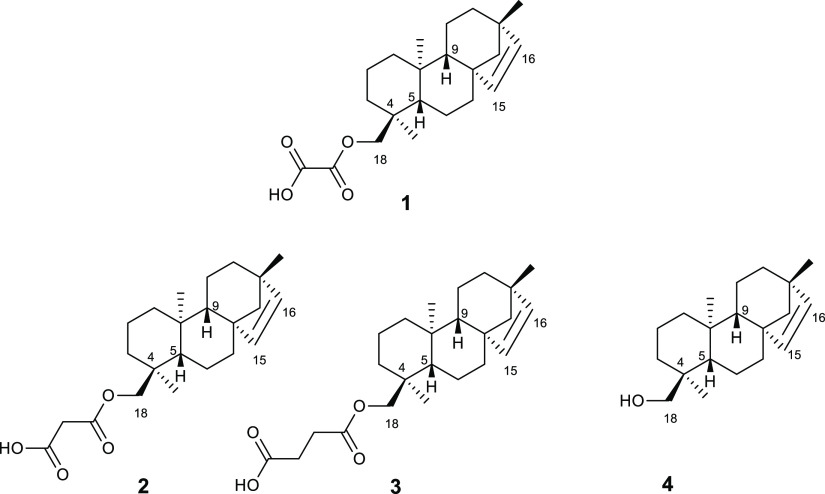
Chemical Structures of Diterpenes **1**–**4** Isolated from *F. densa* var. *ramulosa*

Compound **1** is a tetracyclic *ent*-beyerene diterpene, which was recently discovered by our group and patented for its novel colistin adjuvant activity.^23^ It was isolated from *F. densa* var. *ramulosa* (Solanaceae), a native shrub of Chile, and, to the best of our knowledge, this compound is not available from other chemical sources than our own *in-house* library.^24,25^ It is the oxaloyl ester of the *ent*-beyer-15-en-18-ol (**4**), which was identified in the same plant along with other diterpene analogues, that is, the malonoyl (**2**) and succinoyl (**3**) esters^25^ (Chart 1). To validate the power of the diterpene scaffold as a key platform for further development of ArnT-mediated colistin resistance inhibitors with improved activity, a large variety of chemical analogues was produced for SAR studies. In particular, different derivatives of compound **1** were synthesized with the aim to investigate the role of (i) the length and flexibility of the alkyl chain of the functional group at C-18; (ii) the chirality of C-4; (iii) the presence of a sugar unit to mimic L-Ara4N; and (iv) the unsaturation between C-15 and C-16, on the biological properties of the original diterpene scaffold. To assess whether the expansion of the alkyl chain between the carbonyl groups as well as its removal at C-18 could affect the colistin adjuvant activity, the analogues **2**–**4** were repurposed and some of them were prepared according to the semisynthetic procedure previously described by using alcohol **4** as the starting material.^25^ However, the semisynthetic approach based on the employment of **4** has important limitations: (i) its low concentration in the Chilean plant *F. densa* var. *ramulosa*; (ii) the need of multistep purification of the raw material, and (iii) a restricted number of feasible chemical modifications. Therefore, other natural related scaffolds were evaluated as a source for the starting material. Among them, stevioside **5**, *ent*-kaurenoid diterpene glycosides from *Stevia rebaudiana*, is an ideal candidate, given the easy accessibility to this plant, the low commercial cost, and structural similarity to the molecule of interest.

**Chart 2:**
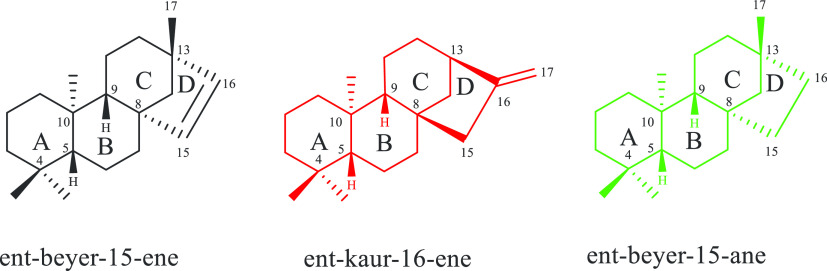
*ent*-Beyerene, *ent*-Kaurene, and *ent*-Beyerane Scaffolds

Importantly, *ent*-kaurene and *ent*-beyerene diterpenes are closely related compounds (Chart 2).

**Scheme 1:**
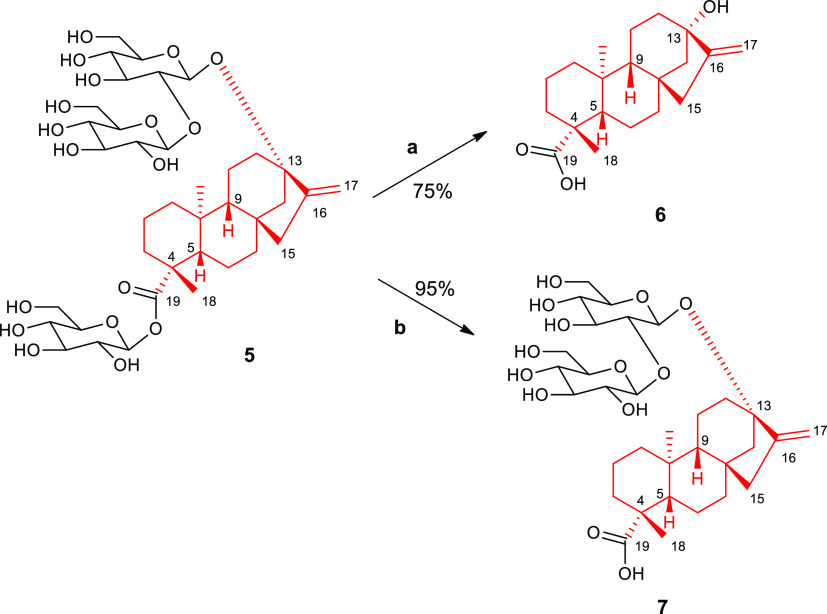
Synthetic Pathway of Compounds **6** and **7** Reagents and conditions: (a) 1. NaIO_4_ in H_2_O, r.t., 16 h, 2. KOH, reflux, 1 h; (b) KOH 10%, 100 °C, 1 h.

They share a common skeleton featuring the “*ent*” configuration, in which the absolute stereochemistry of the A/B ring junction (C-5βH, C-10αMe) is opposite to that of steroids. Both scaffolds are featured by the presence of the bicyclo[3.2.1]octane moiety, a bridged ring system (for C and D rings) attached to C-8 creating a spiro center at this position, and a 1:3-diaxial interaction between the C-10 methyl group and a bridge carbon at C-8. They differ for the double bond between C-15 and C-16, which is exocyclic in *ent*-kaurene scaffold and endocyclic in the *ent*-beyerene one.^26^ Accordingly, the *ent*-kaurene compounds **6** and **7**, known as steviol and steviolbioside, respectively, were synthesized starting from the commercially available stevioside (**5**), which consists of the aglycone steviol 6 (*ent*-13-hydroxykaur-16-en-19-oic acid) and three β-glucopyranosyl moieties at C-19 and C-13 (Scheme 1). As previously reported,^27^ compound **5** was oxidized by using sodium periodate to the corresponding hexaaldehyde, which was further hydrolyzed in a strong alkali environment to yield **6** (75%). Furthermore, compound **7** was obtained by alkaline hydrolysis of **5** in 95% yield.^28^ Besides bearing a different skeleton, these diterpenes feature an opposite configuration at C-4 with respect to compound **1**. Notably, stevioside **5** is widely used to provide a skeletal rearrangement to the *ent*-beyerane core structure of isosteviol (**8**) under acidic conditions.^26,29^

**Figure 1:**
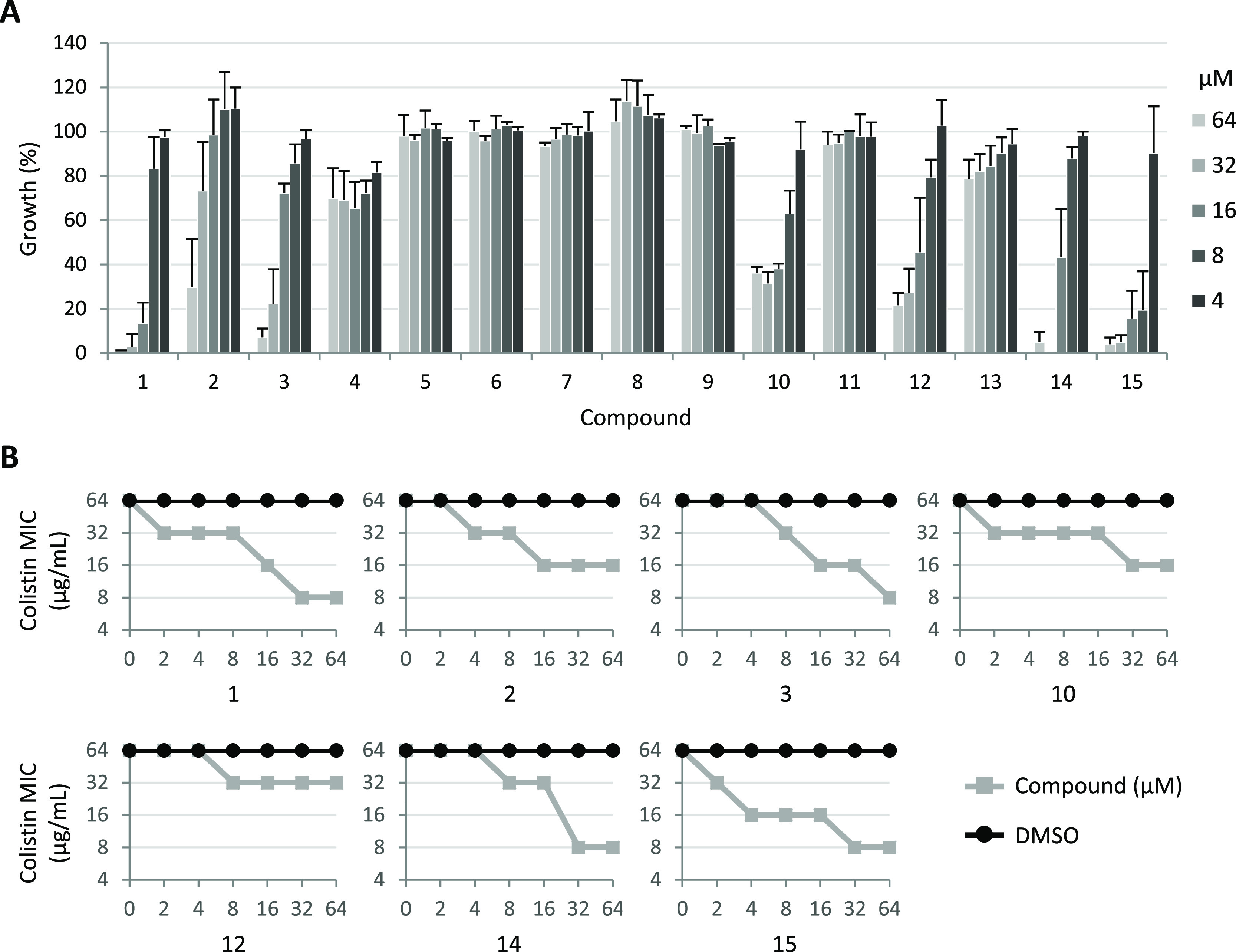
(A) Dose-dependent effect of compounds **1–15** on PA14 col^R^ 5 growth after 24 h at 37 °C in MH supplemented with 8 μg/mL colistin. Growth values are expressed as percentage relative to the cultures treated with equivalent concentrations of DMSO and represent the mean (±SD) of at least three independent experiments. (B) Effect of different concentrations of compounds **1**, **2**, **3**, **10**, **12**, **14**, and **15** on the MIC of colistin for PA14 col^R^ 5 (gray lines) as determined by checkerboard assays. As control, DMSO was used at equivalent concentrations (0.02–0.64%; black lines). The graphs are representative of at least three independent experiments.

**Scheme 2:**
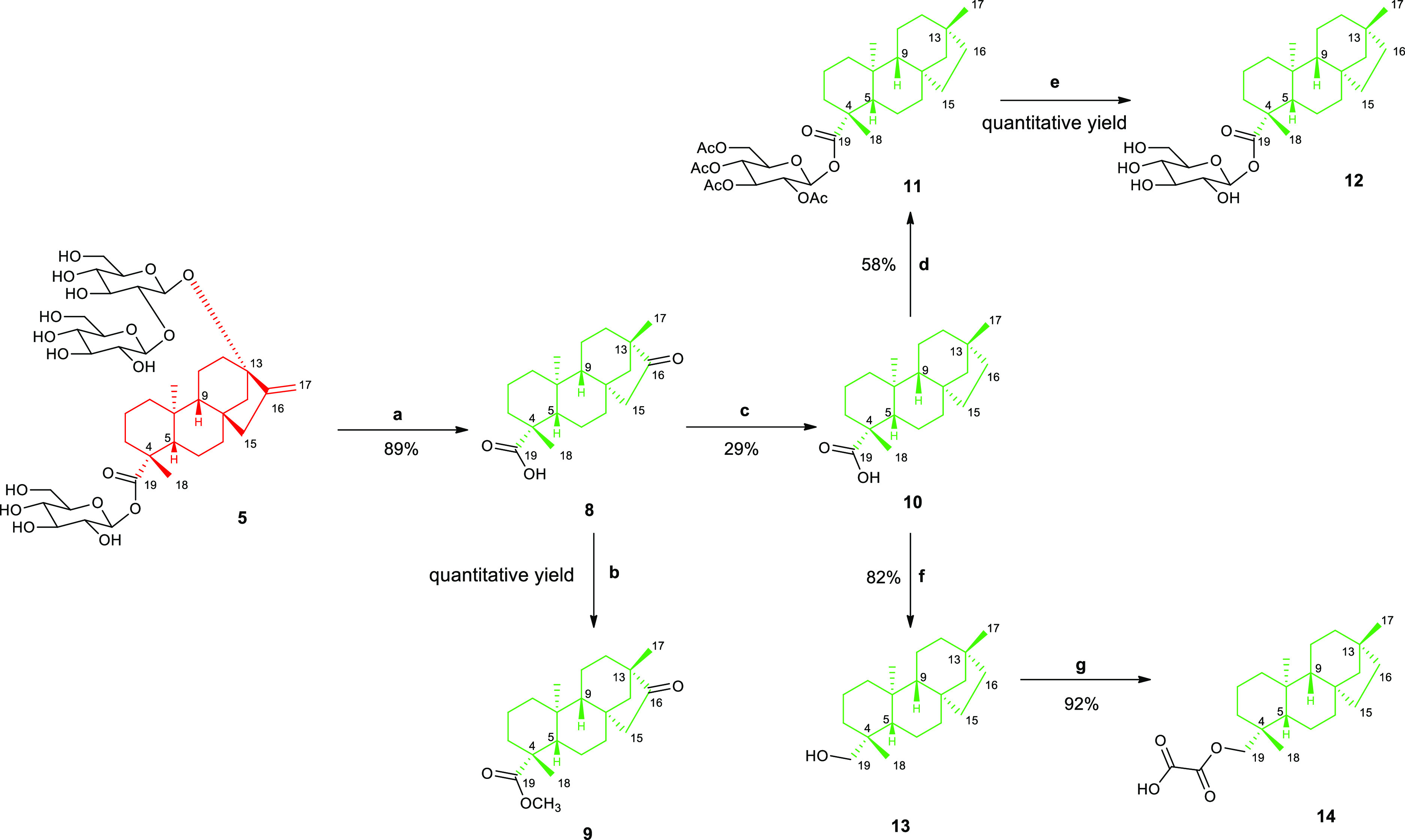
Synthetic Pathway of Compounds **8**–**14** Reagents and conditions: (a) HBr, r.t., 12 h; (b) (1) SOCl_2_, dry DMF, r.t. 2 h; (2) Dry MeOH, Et_3_N, r.t. 2 h; (c) 95% H_2_NNH_2_·*x*H_2_O/KOH, TEG, 150–200 °C, 24 h; (d) peracetylated glucosyl bromide, K_2_CO_3_/TBAB/CH_2_Cl_2_/H_2_O, reflux, 24 h; (e) Et_3_N/MeOH/H_2_O/hexane, r.t. 48 h; (f) LiAlH_4_ in THF 2 M, THF dry, r.t. 3 h; (g) Et_2_O, oxalyl chloride 0 °C → r.t. 30 min.

**Scheme 3:**
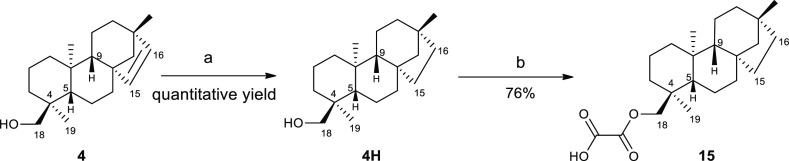
Semisynthesis of Compound **15** Reagents and conditions: (a) H_2_, Pd/C, dry EtOH, r.t., 24 h; (b) Et_2_O, oxalyl chloride, 0 °C → r.t., 30 min.

The *ent*-beyerane skeleton of **8** differs from the parental *ent*-beyerene scaffold for the lack of the unsaturation between C-15 and C-16 and for the absolute configuration at C-4 (R rather than S), thus representing a key platform to create a library of semisynthetic derivatives (**9**–**14**) for SAR studies of diterpene **1**.^30−33^ Treatment of **5** with a strong mineral acid such as hydrobromic acid afforded **8** in 89% yield. This reaction consists of sugar group removal, followed by acid-catalyzed steviol aglycone (**6**) rearrangement and inversion of D ring.^34,35^ In particular, *ent*-kaurene conversion to *ent*-beyerane is an example of Wagner–Meerwein rearrangement, a class of 1,2-rearrangement of carbocation intermediates, which is promoted by the presence of a hydroxyl group adjacent to the 16-alkene.^34,36^ By making chemical transformations at C-16 and C-19 positions of *ent*-beyerane diterpene **8**, compounds **9–14** were further synthesized (Scheme 2). The isosteviol methyl ester **9** was quantitatively obtained by activating **8** in the corresponding acid chloride, followed by the esterification with methanol.^37^ Furthermore, **8** was subjected to the Huang–Minlon modification of the Wolff–Kishner reaction: the carbonyl group at C-16 was reduced to hydrocarbon by strongly heating it with an alkaline solution and hydrazine hydrate and refluxing in an oil bath with triethylene glycol.^38,39^ This reaction led to **10** (yield 29%), namely, isostevic acid, which was then used to synthesize analogues **11**–**14** by chemical modifications of the carboxylic group at C-19. In particular, diterpene **11** was obtained *via* the glycosylation reaction with peracetylated glucosyl bromide in 58% yield by using the phase transfer catalyst tetrabutylammonium bromide (TBAB). Further, deacetylation of sugar hydroxyl groups with triethylamine, followed by alkaline hydrolysis, furnished **12** in quantitative yield, which was designed to mimic the L-Ara4N unit.^40,41^ Compound **13** was prepared by reducing **10** with lithium aluminium to the corresponding alcohol (82% yield).^42,43^ Then, the esterification of the hydroxyl group with oxalyl chloride led to **14** in 92% yield,^44^ differing from **1** by the absence of the double bond at C-15 and C-16 and the opposite configuration at C-4. To further assess the role of the *ent*-beyerene endocyclic double bond of **1** in ArnT inhibition, saturated derivative **15** was prepared. In particular, the catalytic hydrogenation (Pd/C) of the alcohol **4** followed by the esterification reaction between **4H** and oxalyl chloride afforded the oxalate ester **15** in 76% yield (Scheme 3).^25,43^ The chemical identity of all these compounds was confirmed by nuclear magnetic resonance (NMR) spectroscopy and high-resolution mass spectrometry (HRMS) (see the Experimental Section and the Supporting Information). To assess the colistin adjuvant activity of the newly synthesized compounds (**2–15**), we first performed the same screening assay that allowed the identification of compound **1** as a colistin potentiator.^22^ Briefly, a reference *P. aeruginosa* strain evolved *in vitro* toward colistin resistance (PA14 col^R^ 5), characterized by a colistin MIC of 64 μg/mL and that was demonstrated to depend on lipid A aminoarabinosylation for colistin resistance,^45,46^ was cultured in the presence of a fixed, subinhibitory concentration of colistin (8 μg/mL) and different concentrations of each compound of interest (4–64 μM). As control, the effect of the compounds on PA14 col^R^ 5 growth in the absence of colistin was also assessed. Compounds **4–9**, **11**, and **13** had no or only marginal effects on bacterial growth (Figure 1A) and were therefore not further investigated in this work.

In contrast, compounds **2**, **3**, **10**, **12**, **14**, and **15**, featuring hydrogen bonding acceptor and donor groups at C-18, showed some inhibitory activity on PA14 col^R^ 5 cultured in the presence of colistin (Figure 1A), without affecting the bacterial growth in the absence of the antibiotic (Figure S11). This implies that, as for compound **1**, these compounds are able to potentiate the colistin activity. Although compounds **2**, **10**, and **12** caused only a partial inhibition (about 65–80%) of PA14 col^R^ 5 growth, the succinoyl analogue **3** and the most related *ent*-beyerane analogues, **14** and **15** completely inhibited bacterial growth in the presence of 8 μg/mL colistin. Notably, **15** was the only compound that appeared more active than the lead compound **1**, included as the internal reference in the assay (Figure 1A). The potentiating effect of compounds **2**, **3**, **10**, **12**, **14**, and **15** on the colistin activity was further investigated through checkerboard assays and, again, directly compared to that of the lead compound **1**. As shown in Figure 1B, all compounds caused a dose-dependent reduction of colistin MIC for PA14 col^R^ 5, thus confirming that these compounds enhance the colistin activity. In line with the results obtained with the preliminary screening assay (Figure 1A), compounds **2**, **10**, and **12** appeared to be less effective than compound **1**, while the colistin potentiating activity of compounds **3** and **14** was comparable or only slightly lower than that of the lead compound (Figure 1B). Conversely, compound **15**, differing from **1** only by the absence of the double bond at C-15 and C-16, was found to be slightly more active than compound **1**, being able to cause a higher reduction in colistin MIC at low compound concentrations (4–8 μM) (Figure 1B).

To evaluate cytocompatibility of the compounds, with particular focus on lung infections, the bronchial epithelial cell lines 16HBE and CFBE were used, the latter being isolated from a patient with cystic fibrosis homozygous for the F508del CFTR mutation.^47^ Cells were incubated for 18 h with the compounds (range of concentration from 125 to 1.95 μM), and the viability was determined by the 3-[4,5-dimethylthiazol-2-yl]-2,5-diphenyl tetrazolium bromide (MTT) assay.^48^ Collectively, none of the compounds caused substantial reduction of cell viability, in both cell lines, as compared to cells treated with vehicle only (Figure S13). In particular, statistical analysis confirmed that cell viability did not differ significantly between compound-treated and vehicle-treated cells with only very few exceptions, in which some compounds caused a very slight increase of cell viability (Tables S1 and S2). Accordingly, in CFBE cells, compound **1** increased cell viability to 103 and 110% at 62.5 and 15.62 μM, respectively, whereas compounds **2** and **3** increased 16HBE viability up to 105 and 109% at 125 and 31.25 μM, respectively.

**Figure 2:**
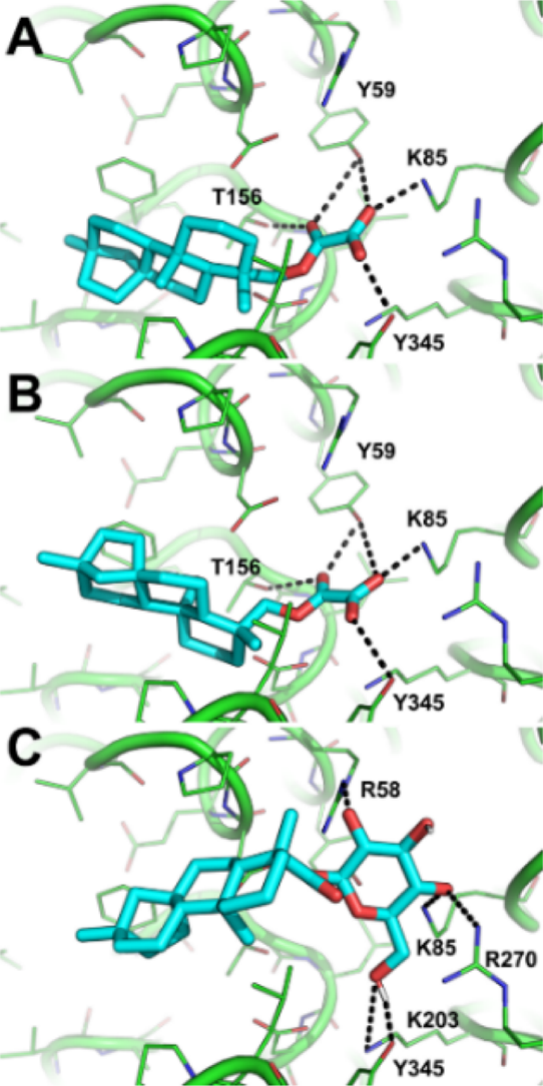
Predicted binding mode of compounds **15** (A), **14** (B), and **12** (C). The crystallographic structure of ArnT coded by PDB ID: 5F15 is shown as green lines and cartoon. Small molecules are shown as cyan sticks. H-bond interactions are highlighted by black dashed lines, while residues contacted by the ligands are labeled.

**Figure 3:**
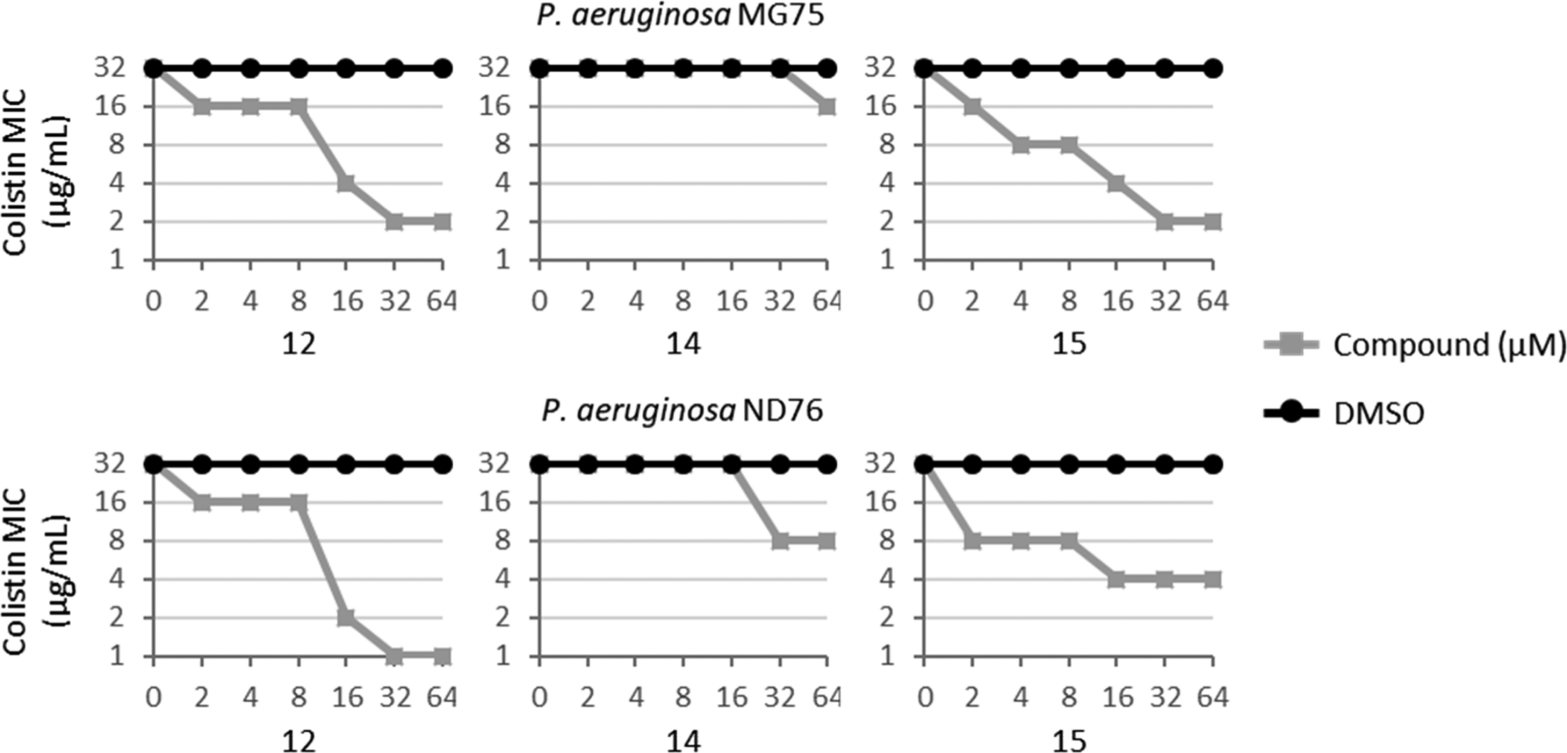
Effect of different concentrations of compounds **12**, **14**, and **15** on the MIC of colistin against two colistin-resistant *P. aeruginosa* clinical isolates as determined by the checkerboard assay. DMSO at the equivalent concentration was used as control (black line). Data are representative of four independent experiments.

The molecular docking simulations of all designed derivatives of compound **1** were carried out with FRED (OpenEye scientific software)^49^ using the crystallographic structure of bacterial ArnT in complex with undecaprenyl phosphate^15^ as the rigid receptor (Figure S14). Whether the Chemgauss4 function was unable to discriminate between active and inactive compounds of this series, rescoring with the XSCORE function,^50^ highlighted **12**, **14**, and **15** as the highest affinity ligands for ArnT in agreement with preliminary biological results (Table S3), thus facilitating further computational design approaches. The docking protocol already adopted in the study of the parent compound **1** was used herein. Compared to the previous study,^22^ in these simulations, only one predominant pose of the compounds was observed. The results are highly comparable to those obtained for **1** and show that the polar moiety (i.e., the oxalyl group in **14** and **15** and the sugar in **12**) binds the hydrophilic cavity that accommodates the phosphate group of the cocrystalized ligand (Figure 2), which was predicted as a putative position for the aminoarabinose sugar substrate of ArnT.^15^ In more detail, **14** and **15** share a very similar binding mode, with the oxalyl group establishing H-bond interactions with Y59, K85, T156, and Y345. The sugar group of derivative **12** is H-bonded to R58, K85, K203, K270, and Y345. Notably, these residues are highly conserved and crucial to the function of the ArnT enzyme. The diterpene group binds in the lipophilic cavity that is accessible from the outer leaflet of the inner membrane and has been proposed to accommodate the alkyl chains and the glucosamine sugar backbone of lipid A. According to the biological results on the reference strain PA14 col^R^ 5 and to the computational data, compound **12**, featuring a sugar moiety at C-19 that mimics L-Ara4N, and the almost stackable *ent*-beyerane analogues, **14** and **15**, were also tested against two colistin-resistant *P. aeruginosa* clinical isolates, that is, *P. aeruginosa* MG75 and ND76.^22^ As reported in Figure 3, the checkerboard assay showed that the three compounds retained the potentiating effect on the colistin activity against both strains. In particular, against *P. aeruginosa* MG75, compounds **12** and **15** were found to cause a 16-fold reduction of colistin MIC, when used at 32 and 64 μM. Even if less marked, the ability of the compounds to decrease the MIC of colistin was manifested also at concentrations ≤16 μM.

**Figure 4:**
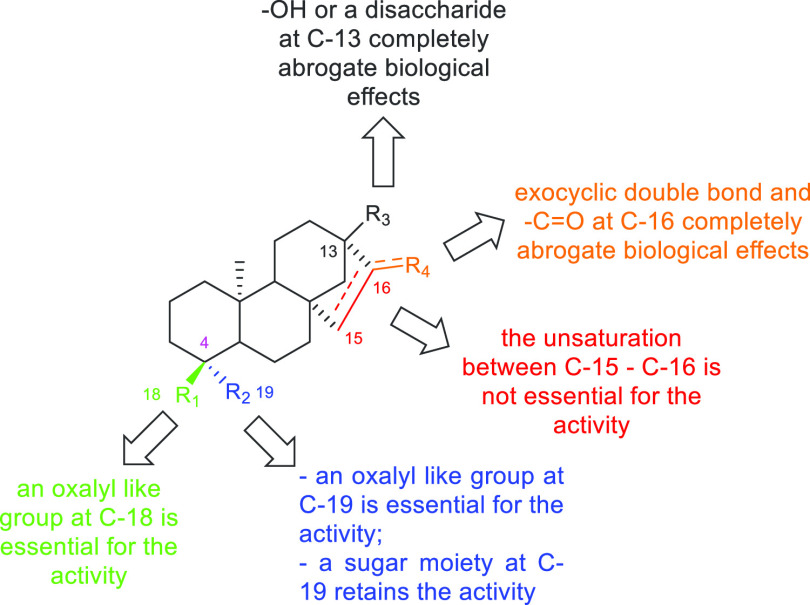
Structure–activity relationships of **1** and its analogues.

In comparison, compound **14** led to a twofold decrease of colistin MIC at 64 μM. A similar trend was obtained against *P. aeruginosa* ND76 at a compound concentration ranging from 2 to 16 μM, while at the highest dosages, compounds **12**, **14** and **15** lowered the MIC of colistin by 32-, 4-, and 8-fold, respectively. Note that as found for PA14 col^R^ 5 (Figure S11), compounds **12**, **14**, and **15** did not affect the bacterial growth of the two bacterial clinical isolates MG75 and ND76 (Figure S12). Overall, the whole biological data coupled with *in silico* studies within the catalytic site of ArnT confirmed that a functional group at C-18 (C-19 for analogues obtained from *ent*-kaurene scaffold) able to establish H-bond interactions within the binding site is essential and suggested that the C-4 stereochemistry probably assists the correct orientation of the same group in the binding pocket. Other diterpene analogues showed no effect as colistin adjuvants. In particular, in the case of *ent*-kaurene analogues, SAR studies indicated that a carboxyl acid or the ß-d-glucose ester at C-19 as well as a hydroxyl group or a 2-O-β-d-glucose-β-d-glucose moiety at C-13 completely abrogate biological effects. The thorough analysis of the colistin adjuvant activity related to *ent*-beyerene scaffold (Figure 4) indicated that (i) the *ent*-beyerane scaffold has a higher activity than the *ent*-beyerene one, suggesting that the unsaturation between C-15 and C-16 is not crucial for such an activity; (ii) an oxalate-like group at C-18 or at C-19 is essential for the activity; (iii) the length and flexibility of the alkyl chain of the functional group at C-18 or at C-19 affect the biological activity; while (iv) the presence of a sugar moiety at C-19 retains the activity likely mimicking L-Ara4N. The computational results are fully consistent with the hypothesis of ArnT inhibition as a mechanism to potentiate the colistin activity against colistin-resistant *P. aeruginosa* strains, strengthening the therapeutic potential of *ent*-beyerane diterpenes as novel colistin adjuvant agents without inherent cytotoxicity.

Remarkably, only a few examples of colistin adjuvants have been reported to date. Barker and co-workers have recently explored commercially available kinase inhibitor libraries and identified IMD-0354 as an effective adjuvant to disarm colistin resistance in Gram-negative bacteria. However, unlike our case, only a limited efficacy against *P. aeruginosa* was observed.^51,52^

The same author also identified derivatives of tryptamine capable to overcome colistin resistance in Gram-negative bacterial pathogens but without investigating their effect against *P. aeruginosa*.^53^

## Conclusions

In summary, we have designed, synthesized, and tested a library of semisynthetic analogues for exploring SAR of diterpene **1**. Considering the overall outcome of microbiology and computational investigations, it can be asserted that compounds able to interact with the catalytic site of ArnT are a privileged tool for an efficient reversal of colistin-resistant strains to its susceptibility. Remarkably, we have demonstrated, for the first time, that an *ent*-beyerane scaffold bearing an oxalate-like group at C-18/C-19 or a sugar residue at C-19 to resemble L-Ara4N is an essential requirement for a more efficient inhibition of bacterial growth likely resulting from a more efficient inhibition of the ArnT activity. Importantly, the easy accessibility of the *ent*-beyerane scaffold from *S. rebaudiana* secondary metabolites will provide a cost-effective key platform for the development of promising colistin resistance inhibitors.

## Experimental Section

### General Methods and Instrumentation

All nonaqueous reactions were performed under an argon atmosphere using flame-dried glassware and standard syringe/septa techniques. All absolute solvents were purchased from Sigma-Aldrich and were of anhydrous grade and used without further purification unless otherwise stated. Solvents for extractions, flash column chromatography (FC), and thin-layer chromatography (TLC) were purchased from Sigma-Aldrich and were of commercial grade and used without further purification unless otherwise stated. The reactions were magnetically stirred and monitored by TLC performed on Merck TLC aluminum sheets (silica gel 60 F254). Spots were visualized with UV light (λ = 254 nm). Chromatographic purification of products (FC) was performed using Sigma-Aldrich silica gel 60 for preparative column chromatography (particle size 40–63 μm). Melting points (Mp) were obtained in open capillary tubes using a Büchi melting point apparatus B-545 and are uncorrected. ^1^H NMR and ^13^CNMR spectra were recorded in CDCl_3_, acetone-*d*_6_, DMSO-*d*_6_, or methanol-*d*_4_ on a Bruker AV-400 400 MHz spectrometer (operating at 400 MHz for ^1^H and 100 MHz for ^13^C) at room temperature and tetramethylsilane (TMS) as the internal standard. Chemical shifts (δ) are reported in parts per million (ppm) and are referenced to CDCl_3_ (δ = 7.26 ppm for ^1^H, δ = 77.16 ppm for ^13^C), acetone-*d*_6_ (δ = 2.05 ppm for ^1^H, δ = 29.84 ppm for ^13^C) DMSO-*d*_6_ (δ = 2.50 ppm for ^1^H, δ 39.52 ppm for ^13^C), or MeOH-*d*_4_ (δ = 3.31 ppm for ^1^H, δ 49.00 ppm for ^13^C). All ^13^C NMR spectra were measured with complete proton decoupling. Data for NMR spectra are reported as follows: s = singlet, d = doublet, t = triplet, q = quartet, m = multiplet, br = broad signal, *J* = coupling constant in Hz. High-resolution mass spectra (HRMS) were recorded on Bruker BioApex Fourier transform ion cyclotron resonance (FT-ICR) mass spectrometer. Mass spectra (MS) were recorded on BRUKER Esquire 3000 PLUS (Esi Ion Trap LC/MSn System).

### Synthesis and Characterization of Compounds **1–4**

Compounds **1**, **2**, **3**, and **4** were isolated and synthesized according to the procedure previously reported and the chemical structures were confirmed based on reported data.^24,25^

### Synthesis of Compound *ent*-13-Hydroxykaur-16-en-19-oic Acid (**6**)

A solution of **5** (1.36 mmol, 1.1 g) and NaIO_4_ (7 mmol, 1.5 g) in water (75 mL) was stirred at room temperature for 16 h. Then, KOH (134 mmol, 7.5 g) was added and the reaction mixture was stirred under reflux in an oil bath for 1 h. After that, the mixture was cooled and neutralized with CH_3_COOH. The aqueous layer was extracted with Et_2_O, and the organic layer was washed with water, dried over anhydrous Na_2_SO_4_, and evaporated to dryness under reduced pressure. The residue was crystallized with CH_3_OH, affording compound **6** (1.02 mmol, 324.58 mg, 75%).^27^ The chemical structure of compound **6** was confirmed based on the reported data.^54^

### Synthesis of Compound 13-[(2-*O*-β-d-Glucopyranosyl-β-d-glucopyranosyl)oxy]-*ent*-kaur-16-en-19-oic (**7**)

A solution of 5 (0.62 mmol, 500 mg) in 10% aqueous KOH (12.5 mL) was stirred at 100 °C in an oil bath for 1 h. Then, the reaction mixture was cooled down, neutralized with a solution of CH_3_COOH 1 N, and evaporated to dryness under reduced pressure. The residue was crystallized with CH_3_OH, yielding compound **7** (0.589 mmol, 390 mg, 95%).^28^ The chemical structure of compound **7** was confirmed based on reported data.^55^

### Synthesis of Compound *ent*-16-ketobeyeran-19-oic Acid (**8**)

Compound **5** (Sigma-Aldrich 260-975-5) (8.69 mmol, 7.0 g) was dissolved in 21 mL of hydrobromic acid (HBr 48% in water), and the dark reaction mixture was stirred for 12 h at room temperature. Then, the precipitate was filtered and solubilized with AcOEt. The organic layer was washed with water and brine, dried over anhydrous Na_2_SO_4_, and evaporated to dryness under reduced pressure. The residue was crystallized with CH_3_OH, yielding compound **8** (7.69 mmol, 2.45 g, 89%).^31^

The chemical structure of compound **8** was confirmed based on reported data.^31,34^

### Synthesis of Compound Methyl *ent*-16-Ketobeyeran-19-oate (**9**)

To compound **8** (0.942 mmol, 300 mg) cooled in an ice bath, SOCl_2_ (13.46 mL) and anhydrous DMF (0.3 mL) were added. The reaction mixture was stirred at room temperature for 2 h and then evaporated to dryness under reduced pressure. The residue was dissolved in anhydrous CH_3_OH (55.4 mL), and Et_3_N (13.46 mL) was added. The solution was stirred at room temperature for 2 h and evaporated to dryness under reduced pressure. The residue was dissolved in CH_2_Cl_2_, and the organic layer was washed with brine three times and dried by anhydrous Na_2_SO_4_ overnight. After filtration, the solution was evaporated to dryness under reduced pressure, giving compound **9** (0.941 mol, 313 mg, quantitative yield).^37^

The chemical structure of compound **9** was confirmed based on reported data.^35^

### Synthesis of Compound *ent*-Beyer-15-an-19-oic Acid (**10**)

A mixture of **8** (3.14 mmol, 1.0 g), triethylene glycol (12.5 mL), 95% hydrazine (2.5 mL), and KOH (22.3 mmol, 1.25 g) was distilled at 180 °C in an oil bath until around 1.25 mL was removed. Then, the reaction was stirred under reflux at 200 °C in an oil bath for 22 h, and after the removal of the condenser, the reaction was left for another 2 h at 200 °C. After that, the reaction mixture was cooled down and 162.5 mL of distilled water was added. The solution was neutralized with glacial acetic acid (CH_3_COOH) (1 N), and the precipitate (which formed on acidification) was filtered and dissolved with Et_2_O. The organic layer was washed with water two times, dried over anhydrous Na_2_SO_4_, and evaporated to dryness under reduced pressure, giving compound **10** (0.911 mmol, 277 mg, 29%).^39^ The chemical structure of compound **10** was confirmed based on reported data.^40,56^

### Synthesis of Compound *ent*-Beyer-15-an-19-oic Acid 2,3,4,6-Tetra-*O*-acetyl-β-d-glucopyranosyl Ester (**11**)

To a solution of **10** (0.986 mmol, 300 mg) in CH_2_Cl_2_ (6.73 mL) and water (1.79 mL) TBAB (0.02 mmol, 6.72 mg), K_2_CO_3_ (3.26 mmol, 450 mg) and peracetylated glucosyl bromide (1.36 mmol, 560 mg) were added. The reaction mixture was stirred under reflux at 50 °C in an oil bath for 24 h. Then, the aqueous layer was extracted with CH_2_Cl_2_, and the organic layer was washed with water two times and with brine and evaporated to dryness under reduced pressure, yielding compound **11** (0,572 mmol, 363 mg, 58%).^40,56^ Brown powder (yield 58%); mp 140 °C ± 0.5 °C; [α]_D_ −8.7° (CHCl_3_). ^1^H NMR (CD_3_OD, 400 MHz, 25 °C, TMS): δ (ppm) 5.84 (d, *J* = 8.4 Hz, 1H, H-1′); 5.34 (t, *J* = 9.6 Hz, 1H, H-3′); 5.13–5.05 (m, 2H, H-2′, H-4′); 4.32 (dd, *J* = 12.4 Hz, *J* = 4.8 Hz, 1H, H-6′); 4.07 (dd, *J* = 12.4 Hz, *J* = 2.4 Hz, 1H, H-6″); 4.02–3.98 (m, 1H, H-5′); 2.04 (s, 6H, 2× CH_3_CO); 2.02 (s, 3H, CH_3_CO); 1.98 (s, 3H, CH_3_CO); 1.88–1.40 (m, 17H); 1.20 (s, 3H, CH_3_-17); 1.14–0.97 (m, 5H); 0.94 (s, 3H, CH_3_-18); 0.72 (s, 3H, CH_3_20). ^13^C{^1^H}NMR (CD_3_OD, 100 MHz, 25 °C, TMS): δ (ppm) 177.16, 172.23, 171.5, 171.2, 170.7, 92.5, 74.5, 73.5, 71.7, 69.4, 62.7, 58.5, 58.4, 57.4, 46.1, 45.1, 42.5, 41.1, 41.0, 40.3, 39.2, 39.0, 38.5, 34.8, 29.2, 27.5, 22.9, 21.8, 20.9, 20.6, 20.5, 19.9, 14.4. ESI-HRMS (positive) *m*/*z*: calcd for C_34_H_50_O_11_Na, 657.3245; found, 657.3248 [M + Na]^+^.

### Synthesis of Compound *ent*-Beyer-15-an-19-oic Acid β-d-Glucopyranosyl Ester (**12**)

Et_3_N (10%, 7.6 mL) was added to a solution of **11** in CH_3_OH/H_2_O/hexane (10:2:1). The reaction mixture was stirred at room temperature for 48 h. Further, it was evaporated to dryness under reduced pressure, and the residue was crystallized with Et_2_O at room temperature, yielding compound **12** (0.511 mmol, 238 mg, quantitative yield).^40,56,57^ White powder (quantitative yield); mp 160 °C ± 0.5 °C; [α]_D_ −22.7° (MeOH). ^1^H NMR (CD_3_OD, 400 MHz, 25 °C, TMS): δ (ppm) 5.41 (d, *J* = 8 Hz, 1H, H-1′); 3.83 (dd, *J* = 12 Hz, *J* = 1.6 Hz, 1H, H-6′); 3.69 (dd, *J* = 12 Hz, *J* = 4.4 Hz, 1H, H-6″); 3.40–3.35 (m, 4H, H-2′, H-3′, H-4′, H-5′); 2.20–2.17 (d, *J* = 13.2, 1H, H-3eq); 2.09–2.04 (m, 1H, H-15eq); 1.91–1.36 (m, 14H); 1.21 (s, 3H, CH_3_-17); 1.18–0.97 (m, 5H); 0.94 (s, 3H, CH_3_-18); 0.87 (s, 3H, CH_3_-20). ^13^C{^1^H} NMR (CD_3_OD, 100 MHz, 25 °C, TMS): δ (ppm) 178.2, 95.5, 78.6, 78.6, 74.0, 71.1, 62.4, 59.1, 58.5, 57.5, 46.2, 45.1, 42.7, 41.2, 41.2, 40.3, 39.3, 39.0, 38.5, 34.5, 29.2, 27.6, 22.8, 21.9, 20.0, 14.4. ESI-HRMS (positive) *m*/*z*: calcd for C_26_H_42_O_7_Na, 489.2822; found, 489.2826 [M + Na]^+^.

### Synthesis of Compound *ent*-Beyer-15-an-19-ol (**13**)

To a stirred solution of **10** (1 mmol, 304 mg) in anhydrous THF (0.0854 n/L, 11.70 mL), LiAlH_4_ (9 mmol, 4.5 mL) was added dropwise, and the reaction mixture was stirred under reflux in an oil bath for 3 h. Further, it was cooled down, quenched by the slow addition of EtOAc and saturated aqueous solution of Rochelle’s salt (sodium potassium tartrate), and evaporated to dryness under reduced pressure, removing the excess of THF. After that, the aqueous layer was extracted with EtOAc and dried over anhydrous Na_2_SO_4_, yielding compound **13** (0.82 mmol, 238 mg, 82%).^42,43^ The chemical structure of compound **13** was confirmed based on reported data.^43^

### Synthesis of Compound *ent*-Beyer-15-an-19-*O*-oxalate (**14**)

To a solution of **13** (0.207 mmol, 60 mg, 1 equiv) in Et_2_O (0.192 mmol/mL, 1.08 mL), oxalyl chloride (0.414 mmol, 0.207 mL, 2 equiv) was added dropwise at 0 °C, and the reaction mixture was stirred under reflux in an oil bath for 30 min. Then, the reaction mixture was cooled down and quenched by slow addition of distilled water. The aqueous layer was extracted with Et_2_O, and the organic layer was washed with water two times and with brine, dried over anhydrous Na_2_SO_4_, and evaporated to dryness under reduced pressure. The residue was purified by FC on silica gel, and a mixture of CHCl_3_:CH_3_OH:HCOOH (98:2:1%) was used as the eluent, affording compound **14** (0.190 mmol; 69 mg, 92%).^44^ Pale yellow oil (yield 92%); r.f. 0.3 (CHCl_3_/CH_3_OH/HCOOH 95:4:1); [α]_D_ +14.3° (CHCl_3_). ^1^H NMR (CDCl_3_, 400 MHz, 25 °C, TMS): δ (ppm) 4.52 (d, *J* = 10.8 Hz, 1H, H-19a); 4.10 (d, *J* = 10.8 Hz, 1H, H-19b); 2.01–1.94 (m, 1H, H-3eq) ; 1.75–1.31 (m, 17H); 1.16–1.03 (m, 4H); 1.00 (s, 3H, CH_3_-17); 0.94 (s, 3H, CH_3_-19); 0.93 (s, 3H, CH_3_-20). ^13^C{^1^H} NMR (CDCl_3_, 100 MHz, 25 °C, TMS): δ (ppm) 158.5, 158.1, 71.1, 57.7, 57.1, 57.0, 45.0, 41.5, 40.0, 39.5, 39.4, 37.6, 37.6, 37.3, 36.0, 33.6, 27.4, 27.2, 20.7, 20.3, 18.0, 15.8. ESI-HRMS (negative) *m*/*z*: calcd for C_22_H_33_O_4_, 361.2384; found, 361.2382 [M – H]^−^.

### Synthesis of Compound *ent*-Beyer-15-an-18-ol (**4H**)

A solution of **4** (0.329 mmol, 95 mg) and Pd/C (6.55 mg, 10%) in EtOH dry (16.4 mL) was stirred under a hydrogen atmosphere (10 bar) at room temperature for 24 h. The reaction mixture was filtered, and the solvent was evaporated under reduced pressure, affording compound **4H** (0.327 mmol, 95 mg, quantitative yield).^43^ White powder (quantitative yield); mp 108 ± 0.5 °C; [α]_D_ −4.6 (CHCl_3_). ^1^H NMR (CDCl_3_, 400 MHz, 25 °C, TMS): δ (ppm) 3.40 (d, *J* = 10.8 Hz, 1H, H-18a); 3.10 (d, *J* = 11.2 Hz, 1H, H-18b); 2.03 (m, 1H, H-3eq.); 1.61–1.07 (m, 21H); 0.95 (s, 3H, CH_3_-17); 0.93 (s, 3H, CH_3_-19); 0.75 (s, 3H, CH_3_-20); ^13^C{^1^H} NMR (CDCl_3_, 100 MHz, 25 °C, TMS): δ (ppm) 72.4, 57.7, 56.9, 49.6, 45.0, 41.0, 40.1, 39.4, 39.4, 37.7, 37.6, 35.3, 33.9, 29.8, 27.3, 20.6, 20.0, 17.9, 17.9, 15.7. ESI-MS (positive) *m*/*z*: calcd for C_20_H_34_ONa, 313.26; found, 313.7 [M + Na]^+^.

### Synthesis of Compound *ent*-Beyer-15-an-18-O-oxalate (**15**)

To a solution of **4H** (0.258 mmol, 75 mg, 1 equiv) in Et_2_O (0.192 mmol/mL, 1.34 mL), oxalyl chloride (0.516 mmol, 0.26 mL, 2 equiv) was added dropwise at 0 °C, and the reaction mixture was stirred under reflux in an oil bath for 30 min. Then, the reaction mixture was cooled down and quenched by slow addition of distilled water. The aqueous layer was extracted with Et_2_O, and the organic layer was washed with water two times and with brine, dried over anhydrous Na_2_SO_4_, and evaporated to dryness under reduced pressure. The residue was purified by flash column chromatography on silica gel and eluted with CHCl_3_:CH_3_OH:HCOOH (98:2:1%), affording compound **15** (0.196 mmol; 71 mg, 76%).^44^ Pale yellow oil (yield 76%); r.f. 0.3 (CHCl_3_/CH_3_OH/HCOOH 95:4:1); [α]_D_ −5° (CHCl_3_). ^1^H NMR (CDCl_3_, 400 MHz, 25 °C, TMS): δ (ppm) 4.10 (d, *J* = 10.8 Hz, 1H, H-18a); 3.90 (d, *J* = 10.8 Hz, 1H, H-18b); 2.03 (m, 1H, H-3eq.); 0.96 (s, 3H, CH_3_-17); 1.70–1.07 (m, 21H); 0.93 (s, 3H, CH_3_-19); 0.89 (s, 3H, CH_3_-20). ^13^C{^1^H} NMR (CDCl_3_, 100 MHz, 25 °C, TMS): δ (ppm) 158.5, 157.8, 57.6, 56.8, 50.6, 44.9, 40.8, 40.0, 39.4, 39.1, 37.7, 37.7, 36.9, 35.8, 33.8, 29.8, 27.2, 20.5, 20.4, 17.6, 17.5, 15.6. ESI-HRMS (negative) *m*/*z*: [M – H]^−^ calcd for C_22_H_33_O_4_, 361.2384; found, 361.2381.

## Biological Assays

### Bacterial Isolates

The *P. aeruginosa* strains used in this study were the *in vitro* evolved colistin-resistant strain *P. aeruginosa* PA14 colR 5^46^ and two colistin-resistant clinical isolates, MG75 and ND76, from the sputum of chronically infected CF patients belonging to a strain collection of the CF Center at the G. Gaslini Institute, Genoa (Italy).^22^ Mueller Hinton broth (MH, Difco) was used for all bacterial assays.

### Screening of Putative Colistin Adjuvants

*P. aeruginosa* PA14 col^R^ 5 was precultured in MH until late exponential phase and diluted at a concentration of *ca.* 5 × 10^5^ cfu/mL in fresh MH containing or not 8 mg/L of colistin and increasing concentrations of each compound of interest (or equivalent amounts of DMSO as control) in 96-well microtiter plates (200 μL volume per well). The growth (OD600) was measured in a Victor3V plate reader (PerkinElmer) after 24 h at 37 °C under static condition and expressed as percentage of growth with respect to the control wells containing the equivalent concentration of DMSO (corresponding to 100%). The effect of increasing concentrations of the active compounds on PA14 col^R^ 5 growth was also evaluated without colistin.

### Checkerboard Assay

Fifty microliters each of two-fold serial dilutions of each compound of interest (0–256 μM) and colistin (0–512 mg/L) in MH were perpendicularly dispensed in 96-well microtiter plates, and each well was inoculated with 100 μL of MH containing *P. aeruginosa* PA14 col^R^ 5 at *ca.* 10^6^ cfu/mL and precultured in MH until mid-exponential phase. Microtiter plates were incubated at 37 °C under static conditions, and the bacterial growth (OD600) was measured in a Victor3V plate reader (PerkinElmer) after 24 h. The same procedure was followed for testing the most promising compounds against the colistin-resistant *P. aeruginosa* clinical isolates.

### Cytotoxicity Assay

The cytotoxicity of the compounds was assessed on the bronchial epithelial cell lines 16HBE and CFBE.^47^ Briefly, cells were expanded on coated flasks^58^ and seeded at 3×10^5^ cells/well in 96-well microtiter plates. On the next day, a fresh medium containing the compound, or vehicle control (DMSO), was added to each well (200 μL per well). Two-fold serial dilutions, from 125 to 1.95 μM, of each compound, and equivalent amount of DMSO were tested. The cells were incubated for 18 h, after which 0.5 mg/mL MTT was added and incubation continued for 3 h at 37 °C. The culture supernatant was then discarded, and the intracellular formazan was dissolved in DMSO (100 μL per well). Absorbance at 570 nm (A570) was read using a microtiter plate reader (Bio-Rad NovapathTM microplate reader). The cell viability was expressed as percentage with respect to untreated cells. Statistical analysis was done by using the two-way ANOVA and comparing the cell viability (%) of cells treated with the compounds respect to cells treated with the equivalent concentration of DMSO.

### Molecular Modeling

The ligand ionization state was assigned by QUACPAC (OpenEye Scientific Software) version 2.0.0.3,^21^ while conformational analysis was carried out with Omega (OpenEye Scientific Software) version 3.1.0.3, storing up to 600 conformers.^59,60^ The receptor was prepared as described previously^22^ based on the crystallographic structure of ArnT in complex with undecaprenyl phosphate (PDB ID: 5F15),^15^ while molecular docking simulations were carried out with FRED (OpenEye Scientific Solutions) version 3.3.0.3^22,49^ storing the five top scoring poses of each ligand. Selected docking poses were rescored with XSCORE.^50^

## Supporting Information Available

^1^H NMR and ^13^C NMR spectra of compounds **11–12**, **14**, **4H**, and **15**; biological activity of compounds on bacterial growth and on the viability of bronchial epithelial cells; and molecular modeling (PDF)
