# Supplementary material for: ent-Beyerane Diterpenes as a Key Platform for the Development of ArnT-Mediated Colistin Resistance Inhibitors
Source: J Org Chem. 2020 Jul 27;85(16):10891–901. doi: 10.1021/acs.joc.0c01459 (PMC8009527; doi:10.1021/acs.joc.0c01459)
Supplement: Supplementary file 1 — jo0c01459_si_001.pdf [file jo0c01459_si_001.pdf]

Supporting information

Ent-beyerane diterpenes as a key platform for the development of ArnT-mediated colistin resistance inhibitors

Deborah Quaglio [§†], Maria Luisa Mangoni [§#], Roberta Stefanelli [±,‡], Silvia Corradi [†,¥], Bruno Casciaro [¥] ,Valeria Vergine [†], Federica Lucantoni [±], Luca Cavinato [±] , Silvia Cammarone [†],Maria Rosa Loffredo [#], Floriana Cappiello [#], Andrea Calcaterra [†], Silvia Erazo [§], Francesca Ghirga [¥*], Mattia Mori [^*], Francesco Imperi [‡], Fiorentina Ascenzioni [±] and Bruno Botta [†]

[†] Department of Chemistry and Technology of Drugs, "Department of Excellence 2018−2022", Sapienza University of Rome, P.le Aldo Moro 5, 00185 Rome, Italy

[#] Laboratory affiliated to Pasteur Italia-Fondazione Cenci Bolognetti, Department of Biochemical Sciences, Sapienza University of Rome, P.le Aldo Moro 5, Rome 00185, Italy

[±] Department of Biology and Biotechnology Charles Darwin, Sapienza University of Rome, Laboratory affiliated to Pasteur Italia-Fondazione Cenci Bolognetti, Via dei Sardi 70, 00185 Rome, Italy

[‡] Department of Science, Roma Tre University, Viale Guglielmo Marconi 446, 00146 Rome, Italy

[¥] Center For Life Nano Science@Sapienza, Istituto Italiano di Tecnologia, Viale Regina Elena, 291, 00161 Rome, Italy

[§] Department of Pharmacological and Toxicological Chemistry, Faculty of Chemical and Pharmaceutical Sciences, University of Chile, Santiago, Chile

[^] Department of Biotechnology, Chemistry and Pharmacy, "Department of Excellence 2018−2022", University of Siena, via Aldo Moro 2, 53100 Siena, Italy

Table of Contents

1. ^{1}H and ^{13}C NMR spectra of compounds 11-12, 14, 4H, 15 ………………………………S2
2. Biological activity……………………………………………………………S7
   2.1. Effect of compounds on bacterial growth……………………………S7
   2.2. Effect of compounds on the viability of bronchial epithelial cells…………S8
3. Molecular modeling……………………………………………………………S10

1.^{1}H and ^{13}C NMR spectra of compounds 11-12, 14, 4H, 15

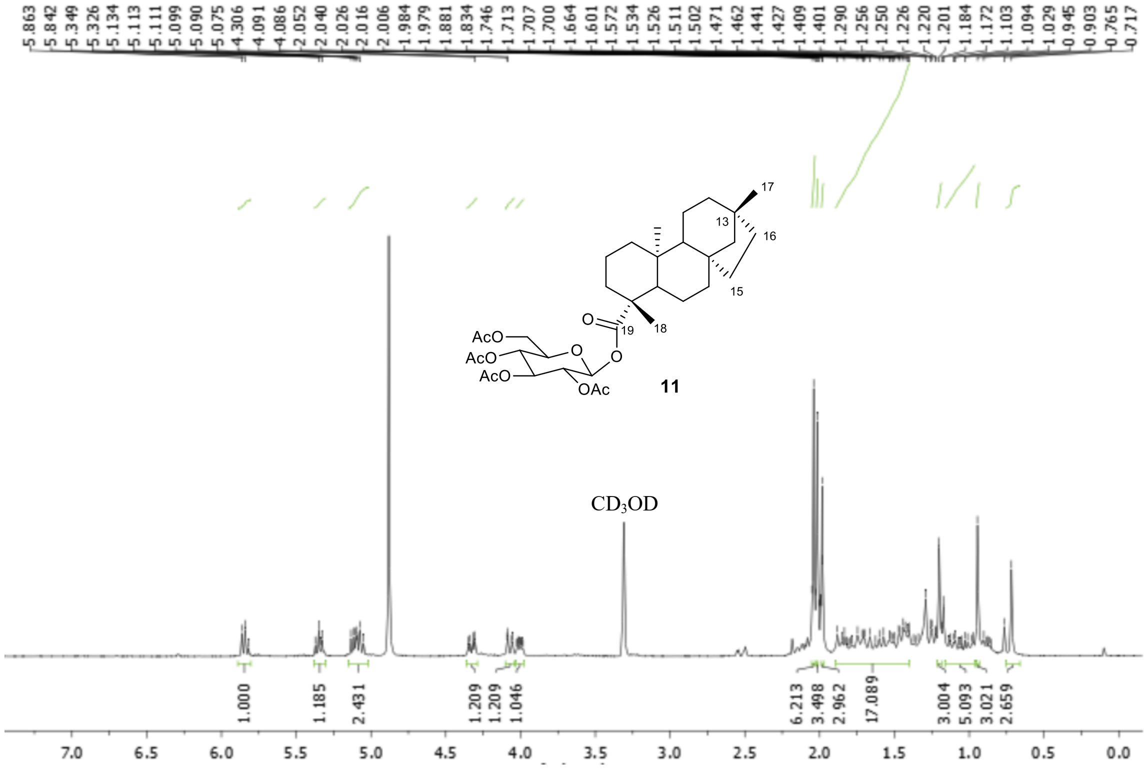

Figure S1. ^{1}H NMR (CD_3OD, 400 MHz) spectrum of compound **11.**

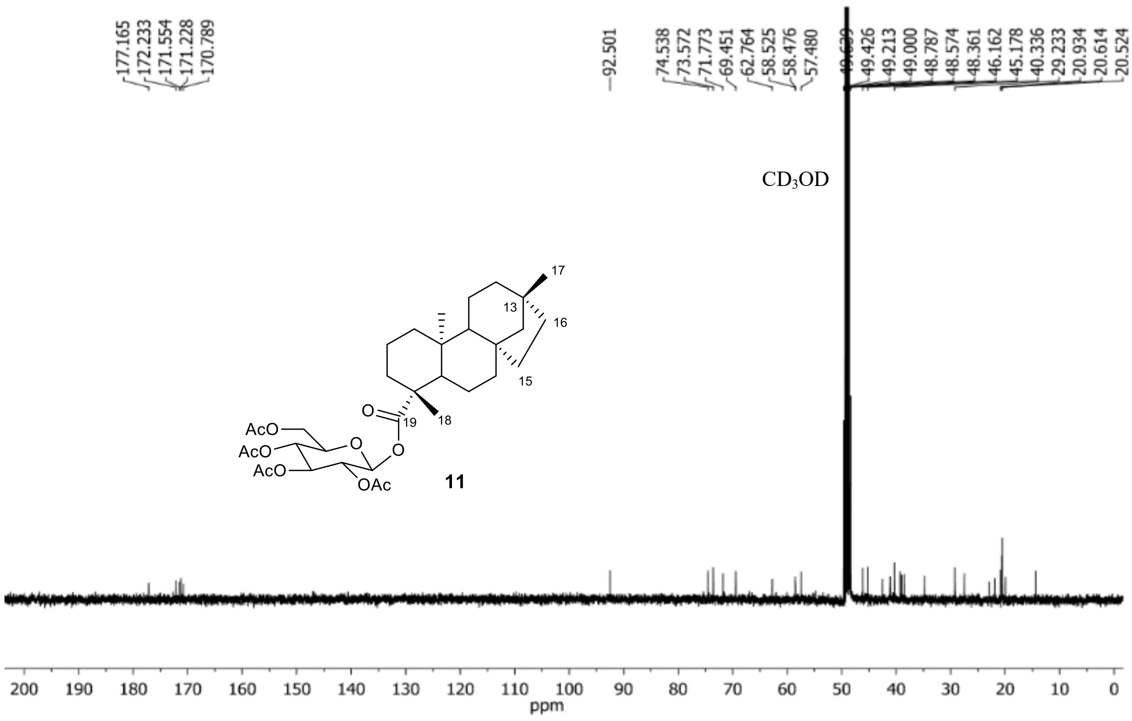

Figure S2. ^{13} C NMR (CD_3OD, 100 MHz) spectrum of compound **11.**

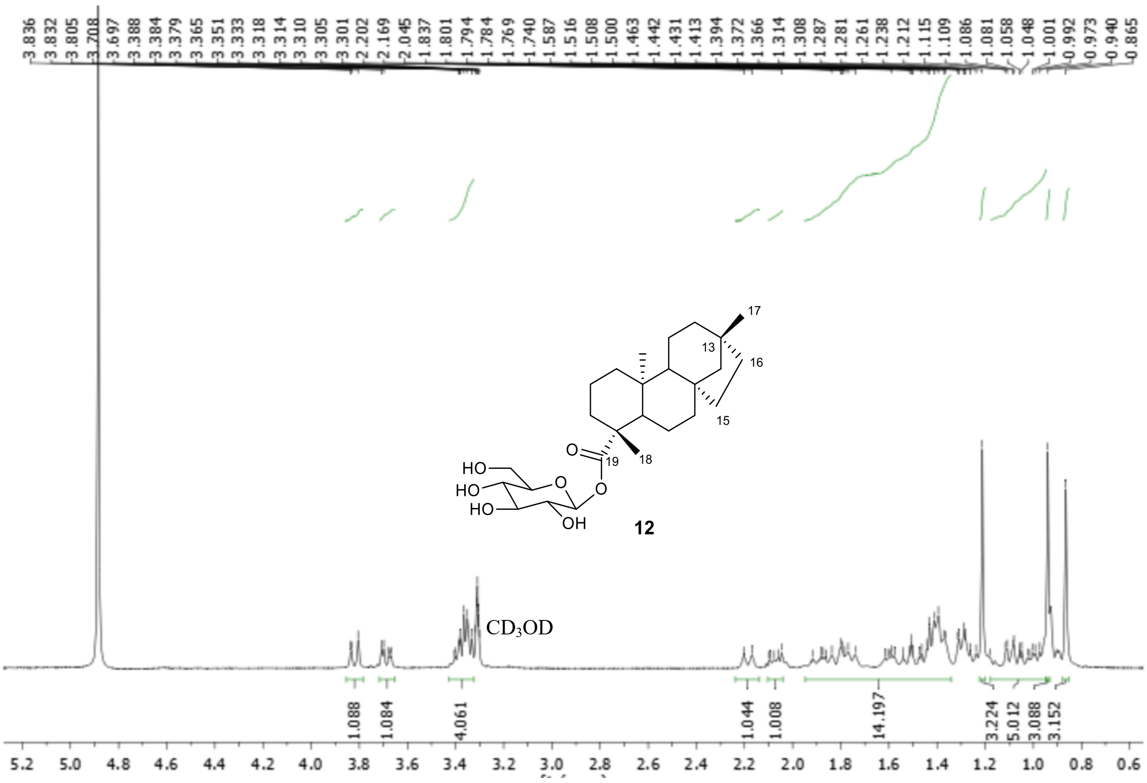

Figure S3. ^{1}H NMR (CD_3OD, 400 MHz) spectrum of compound **12.**

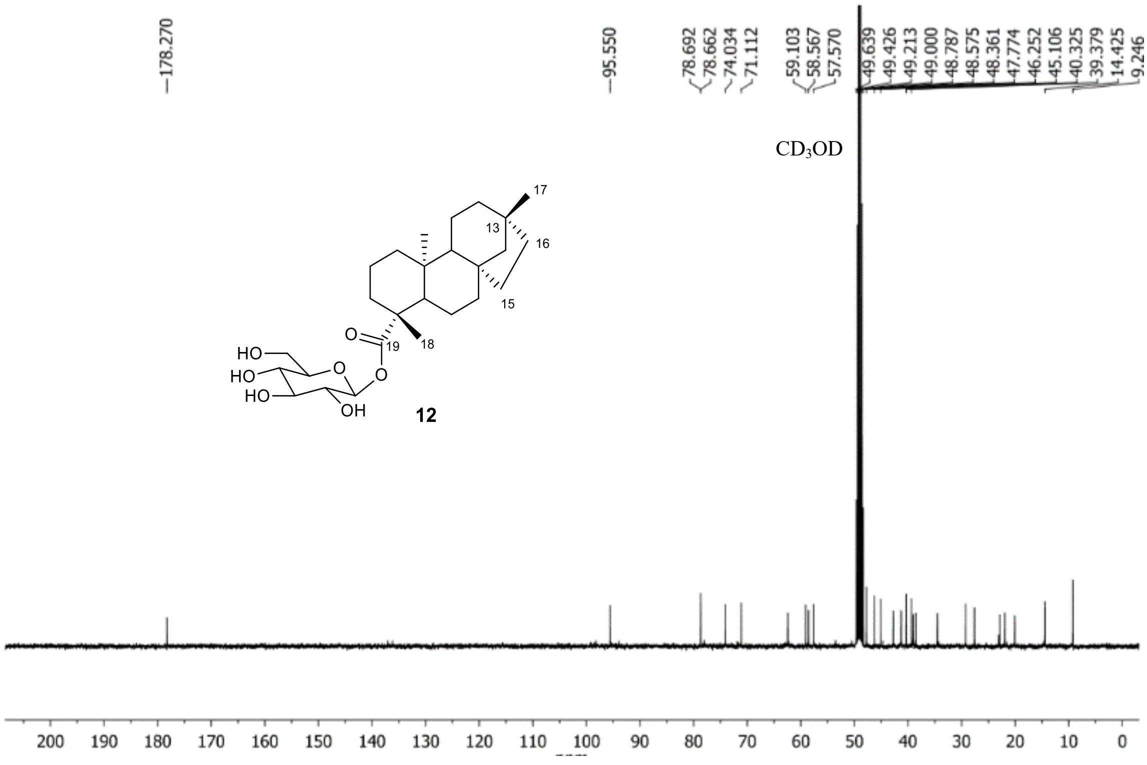

Figure S4. ^{13} C NMR (CD_3OD, 100 MHz) spectrum of compound **12.**

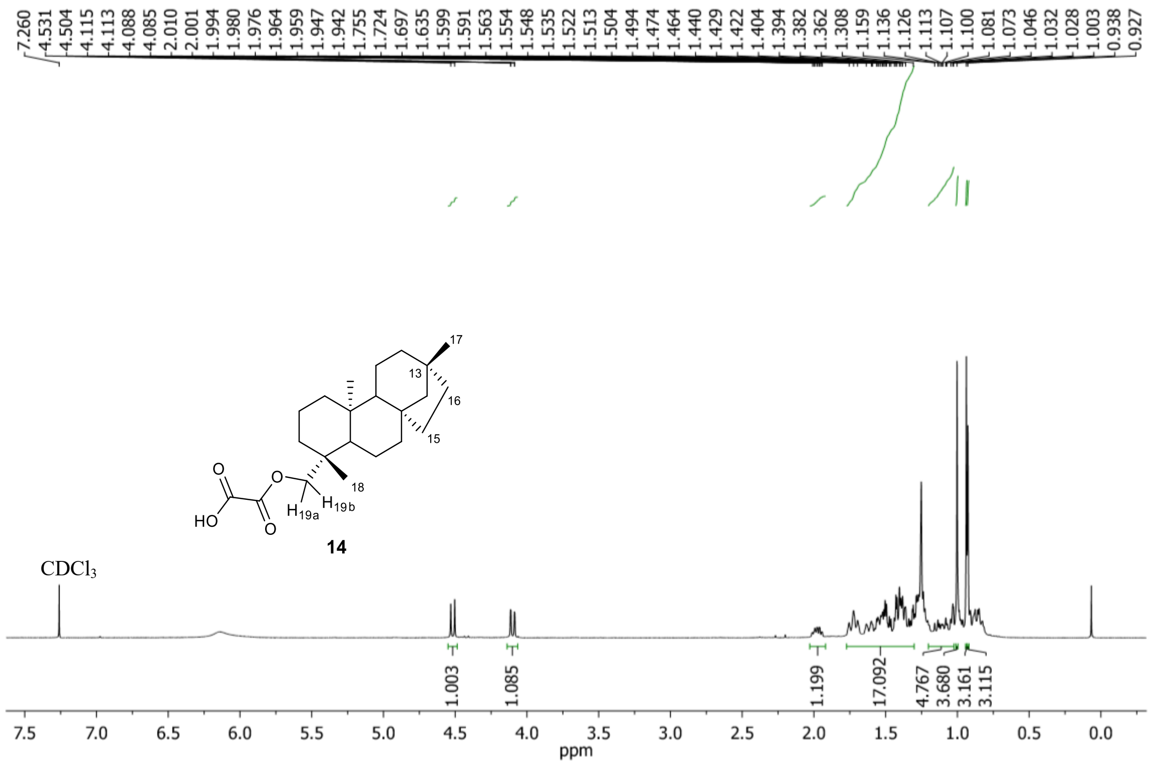

Figure S5. ^{1}H NMR ($CDCl_3$, 400 MHz) spectrum of compound **14.**

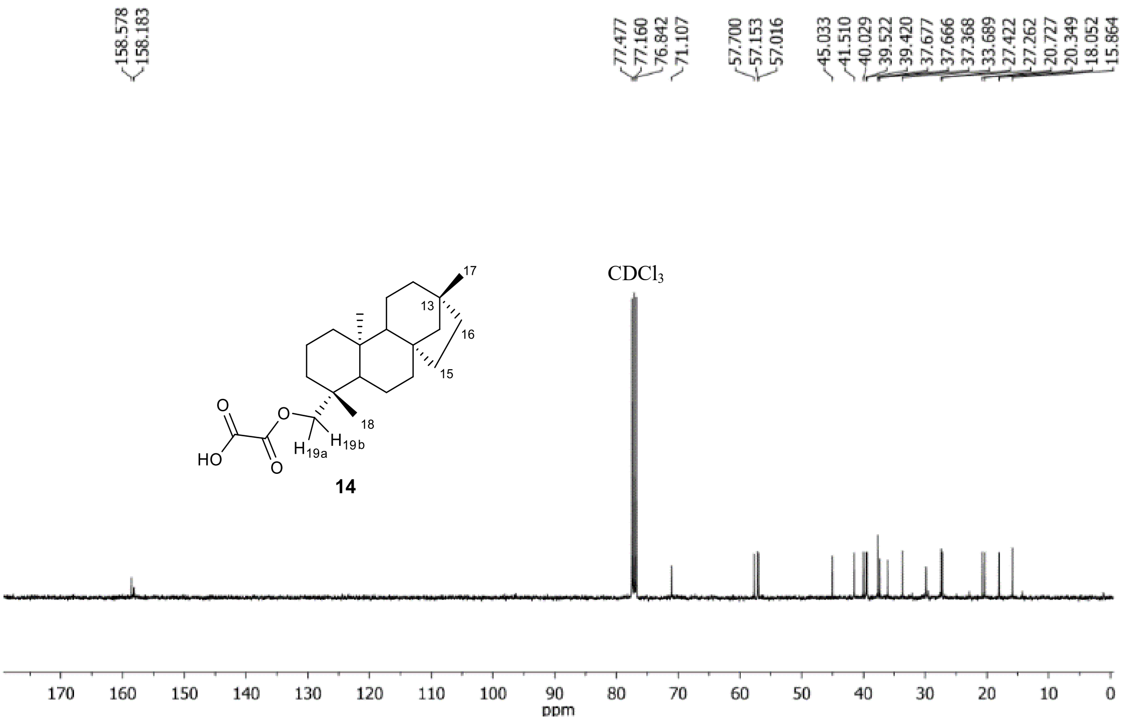

Figure S6. ^{13}C NMR ($CDCl_3$, 100 MHz) spectrum of compound **14.**

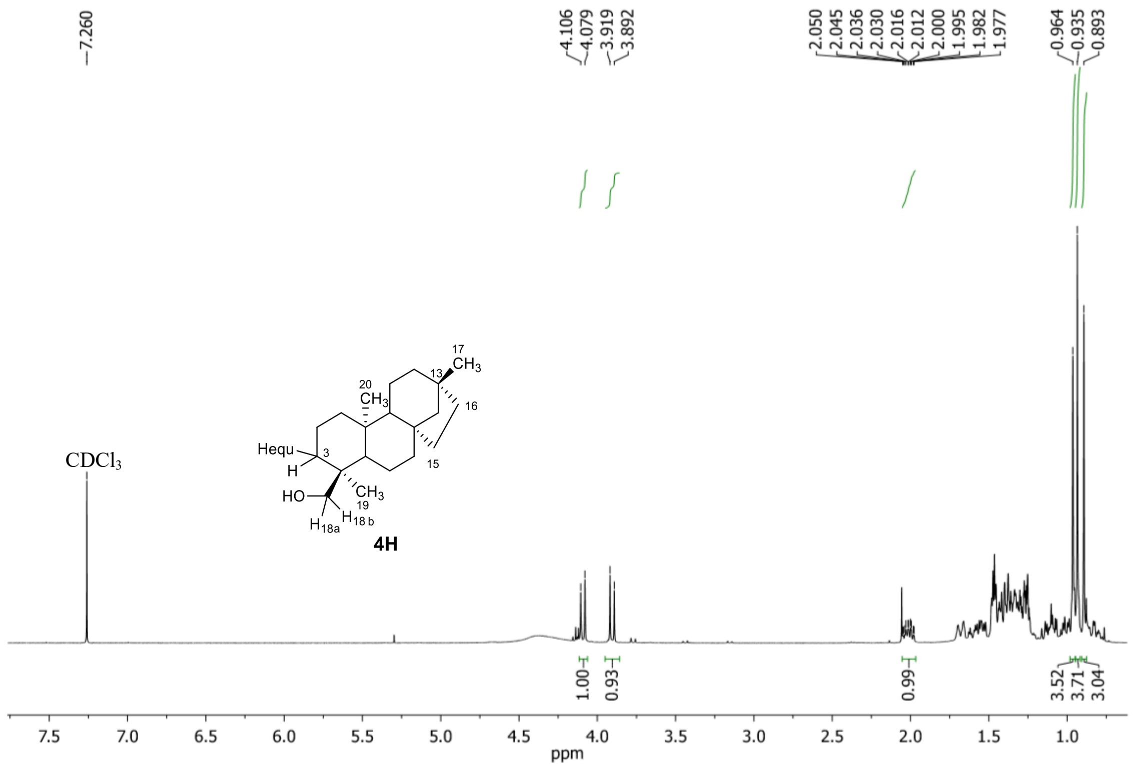

Figure S7. ^{1}H NMR ($CDCl_3$, 400 MHz) spectrum of compound **4H.**

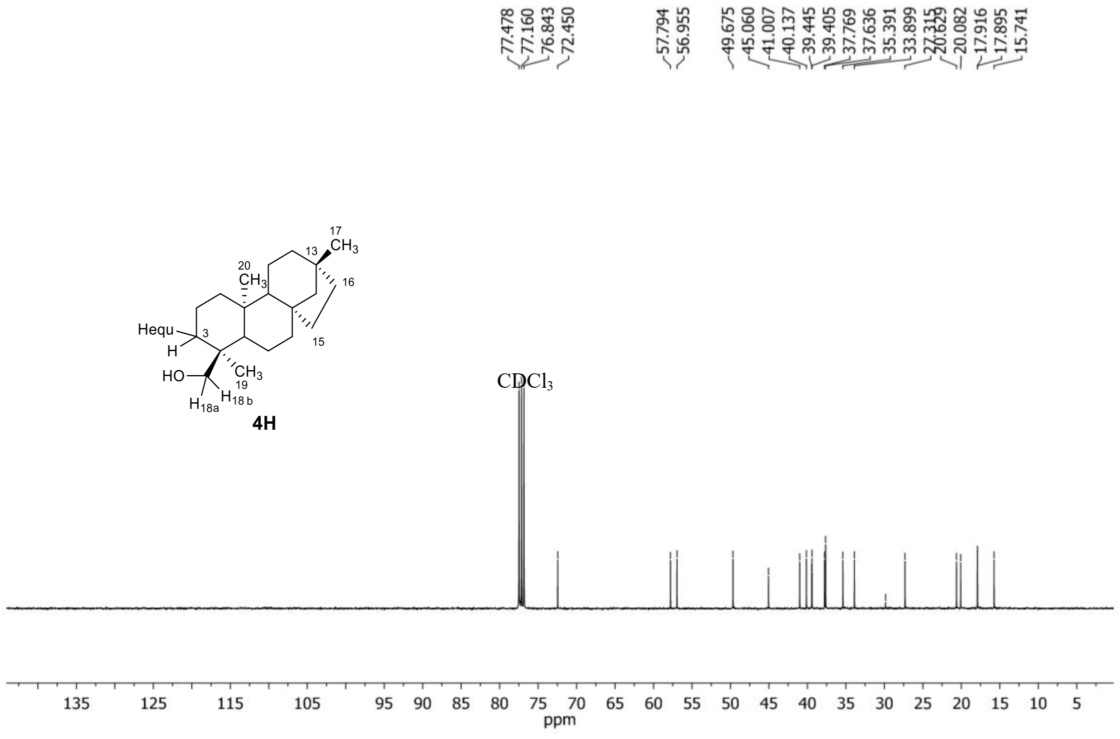

Figure S8. ^{13} C NMR ($CDCl_3$, 100 MHz) spectrum of compound **4H.**

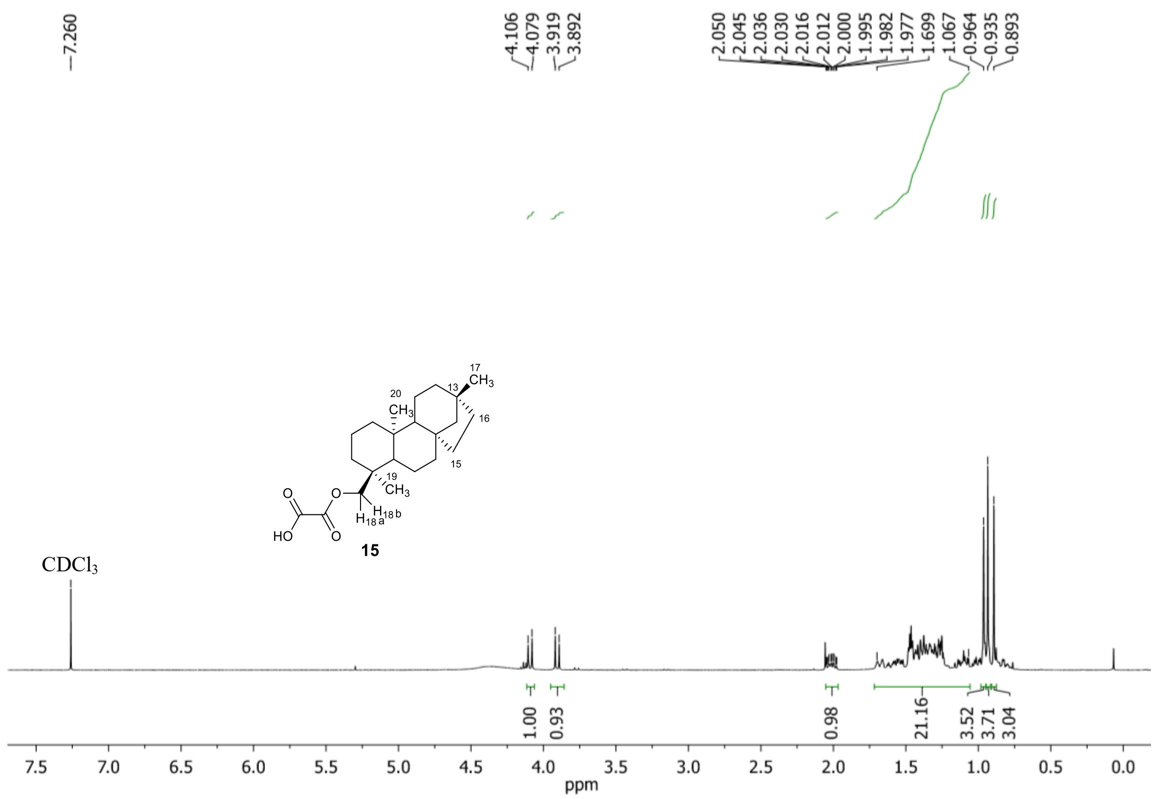

Figure S9. ^{1}H NMR ($CDCl_3$, 400 MHz) spectrum of compound **15.**

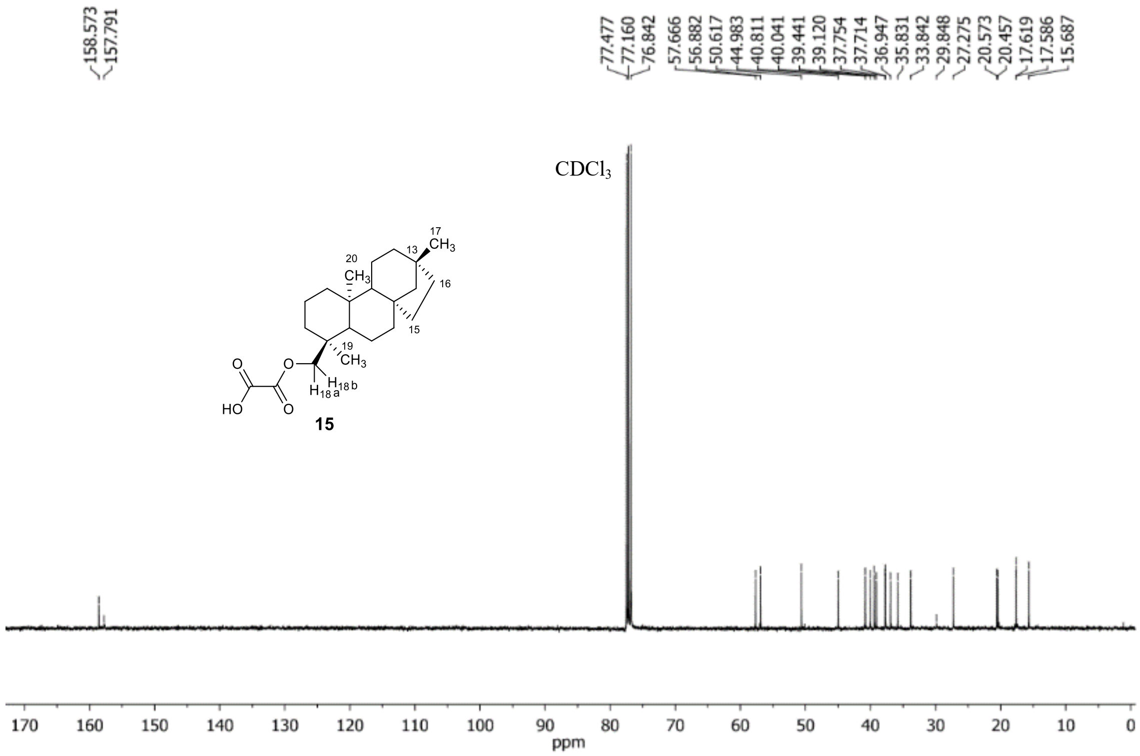

Figure S10. ^{13}C NMR ($CDCl_3$, 100 MHz) spectrum of compound **15.**

2. Biological activity

2.1. Effect of compounds on bacterial growth

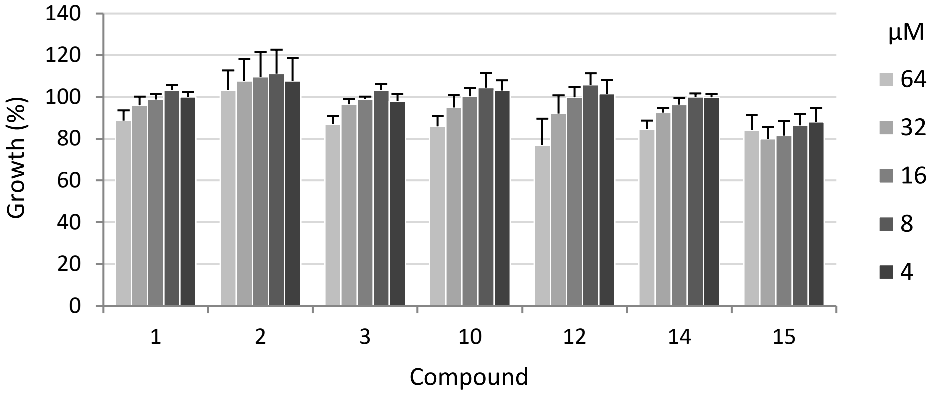

Figure S11. Dose-dependent effect of compounds **1**, **2**, **3**, **10**, **12**, **14** and **15** on PA14 col[R] 5 growth after 24 hours at 37°C in MH (without colistin). Growth values are expressed as percentage relative to the cultures treated with equivalent concentrations of DMSO and represent the mean (±SD) of three independent experiments.

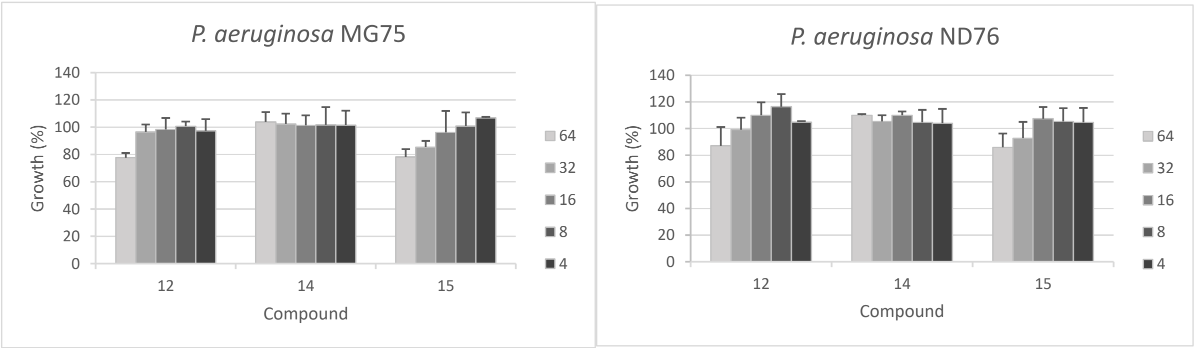

Figure S12. Dose-dependent effect of compounds **12**, **14** and **15** on the growth of the two clinical isolates *P. aeruginosa* MG75 and ND76 after 24 hours at 37°C in MH (without colistin). Growth values are expressed as percentage relative to the cultures treated with equivalent concentrations of DMSO and represent the mean (±SD) of three independent experiments.

2.2. Effect of compounds on the viability of bronchial epithelial cells

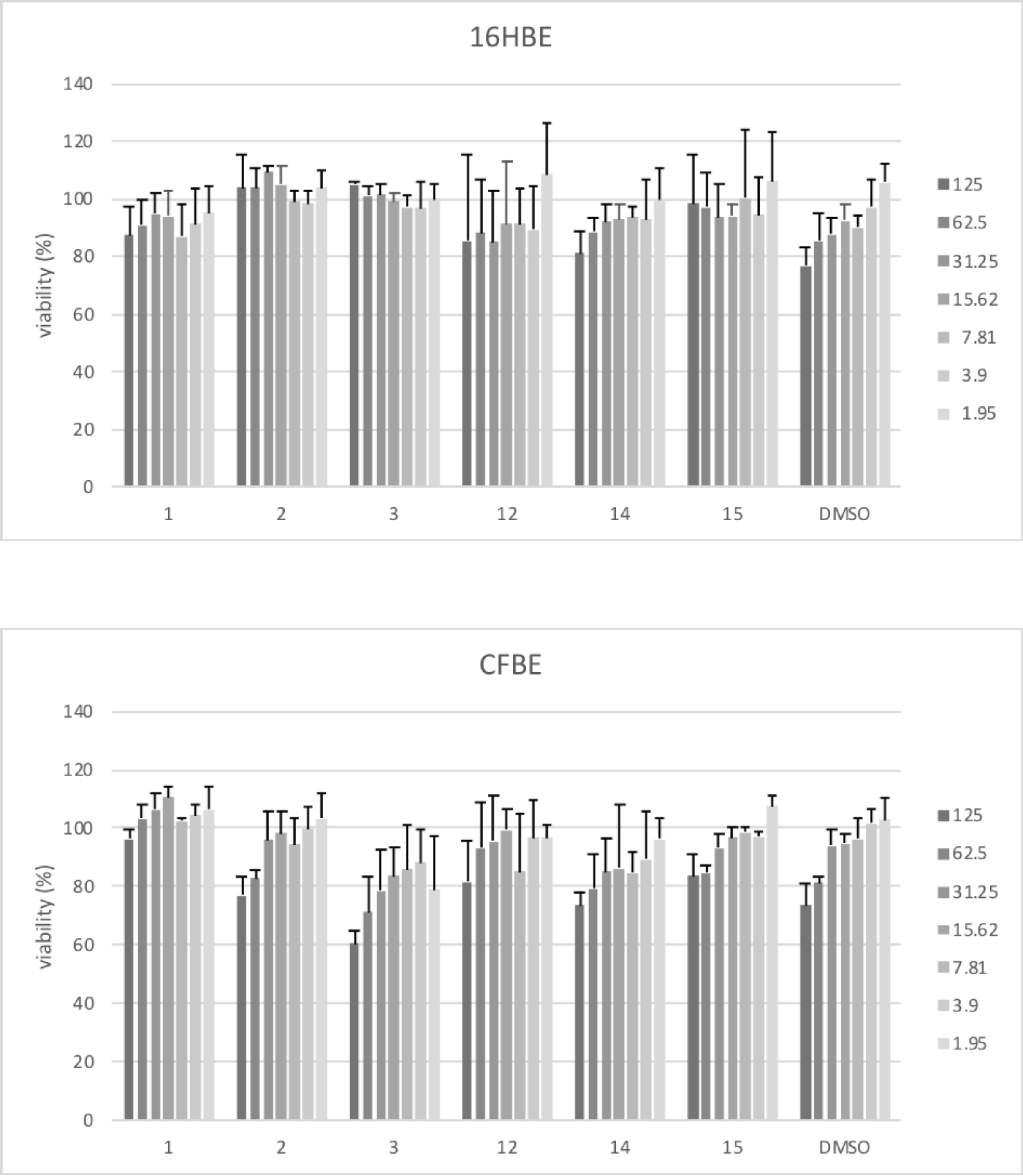

Figure S13. Viability of 16HBE and CFBE epithelial cells exposed to the compounds 1, 2, 3, 12, 14 and 15 at the indicated concentrations, or DMSO at equivalent concentrations, for 18 hours. Cell viability was assessed through the MTT assay and expressed as percentage relative to untreated cells. Data are the mean (±SD) of three independent experiments.

Table S1. *P* values of compound- respect to DMSO-treated 16HBE cells by two-way ANOVA.

μM	1	2	3	12	14	15
125	P=0.5175	P=0.2991	P=0.0374	P=0.0127	P>0.9999	P=0.8276
62.5	P>0.9999	P=0.7566	P=0.0942	P=0.1647	P>0.9999	P>0.9999
31.25	P=0.7531	P>0.9999	P=0.0072	P=0.0283	P>0.9999	P=0.7359
15,62	P>0.9999	P>0.9999	P=0.1173	P=0.3854	P>0.9999	P>0.9999
7.81	P>0.9999	P>0.9999	P=0.0669	P=0.2039	P=0.9870	P=0.4300
3.9	P>0.9999	P>0,.9999	P>0.9999	P>0.9999	P=0.8629	P>0.9999
1.95	P=0.4499	P>0.9999	P>0.9999	P=0.8883	P>0.9999	P=0.7828

Table S2. *P* values of compound- respect to DMSO-treated CFBE cells by two-way ANOVA.

μM	1	2	3	12	14	15
125	P=0.0922	P=0.6880	P>0.9999	P=0.3081	P>0.9999	P=0.0733
62.5	P=0.0401	P=0.5943	P>0.9999	P>0.9999	P=0.6961	P>0.9999
31.25	P=0.2250	P>0.9999	P>0.9999	P=0.7167	P>0.9999	P=0.2424
15,62	P=0.0215	P>0.9999	P>0.9999	P=0.7139	P>0.9999	P=0.6526
7.81	P>0.9999	P>0.9999	P>0.9999	P>0.9999	P=0.3976	P=0.0814
3.9	P>0.9999	P>0.9999	P>0.9999	P=0.6697	P>0.9999	P=0.5528
1.95	P>0.9999	P>0.9999	P>0.9999	P=0.5700	P>0.9999	P>0.9999

3. Molecular modeling

Table S3. XSCORE rescoring results on docking poses predicted with FRED.

Compound	Chemgauss4 [a]	XSCORE [b]
**1**	-3.56	6.35
**2**	-5.14	6.09
**3**	-5.79	6.31
**4**	-2.25	6.29
**5**	-0.22	6.28
**6**	-2.17	6.22
**7**	-1.42	6.15
**8**	-0.46	6.15
**9**	-2.99	6.20
**10**	-4.30	6.26
**11**	1.33	6.20
**12**	-5.51	6.32
**13**	-2.49	6.15
**14**	-6.20	6.63
**15**	-3.21	6.38

[a] – the lower, the better
[b] – expressed as -pK_d, the higher the better

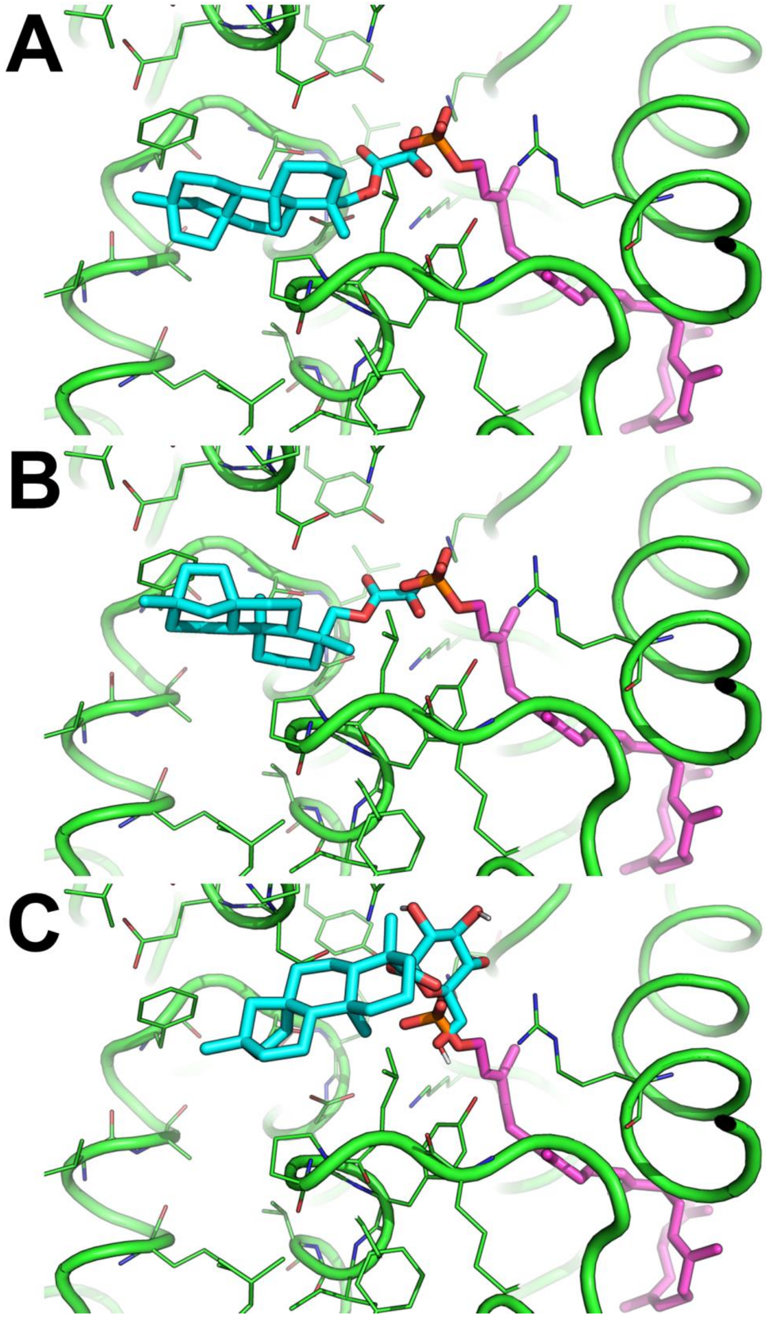

Figure S14. Structural overimposition between the predicted binding mode of compounds **15** (A), **14** (B), and **12** (C) with the crystallographic pose of undecaprenyl phosphate (magenta sticks). The crystallographic structure of ArnT coded by PDB ID: 5F15 is shown as green lines and cartoon. Small molecules studied in this work are shown as cyan sticks.
